# Chimeric Antigen Receptor T-Cells: An Overview of Concepts, Applications, Limitations, and Proposed Solutions

**DOI:** 10.3389/fbioe.2022.797440

**Published:** 2022-06-22

**Authors:** Alaa Alnefaie, Sarah Albogami, Yousif Asiri, Tanveer Ahmad, Saqer S. Alotaibi, Mohammad M. Al-Sanea, Hisham Althobaiti

**Affiliations:** ^1^ Department of Medical Services, King Faisal Medical Complex, Taif, Saudi Arabia; ^2^ Department of Biotechnology, College of Science, Taif University, Taif, Saudi Arabia; ^3^ Department of Clinical Pharmacy, College of Pharmacy, Taif University, Taif, Saudi Arabia; ^4^ Multidisciplinary Centre for Advanced Research and Studies, Jamia Millia Islamia, New Delhi, India; ^5^ Department of Pharmaceutical Chemistry, College of Pharmacy, Jouf University, Sakaka, Saudi Arabia; ^6^ Chief of Medical Department, King Faisal Medical Complex (KFMC), Taif, Saudi Arabia

**Keywords:** chimeric antigen receptor T-cell, adaptive immunity, autoimmune disorder, cancer immunotherapy, solid tumor, tumor infiltration

## Abstract

Adaptive immunity, orchestrated by B-cells and T-cells, plays a crucial role in protecting the body from pathogenic invaders and can be used as tools to enhance the body’s defense mechanisms against cancer by genetically engineering these immune cells. Several strategies have been identified for cancer treatment and evaluated for their efficacy against other diseases such as autoimmune and infectious diseases. One of the most advanced technologies is chimeric antigen receptor (CAR) T-cell therapy, a pioneering therapy in the oncology field. Successful clinical trials have resulted in the approval of six CAR-T cell products by the Food and Drug Administration for the treatment of hematological malignancies. However, there have been various obstacles that limit the use of CAR T-cell therapy as the first line of defense mechanism against cancer. Various innovative CAR-T cell therapeutic designs have been evaluated in preclinical and clinical trial settings and have demonstrated much potential for development. Such trials testing the suitability of CARs against solid tumors and HIV are showing promising results. In addition, new solutions have been proposed to overcome the limitations of this therapy. This review provides an overview of the current knowledge regarding this novel technology, including CAR T-cell structure, different applications, limitations, and proposed solutions.

## 1 Introduction

The global cancer burden, cancer incidence, and mortality estimations have increased rapidly. According to the International Agency for Research on Cancer, 19.3 million diagnosed cases and 10.0 million deaths worldwide in 2020 have been attributed to cancer (Sung et al., 2021). The relationship between cancer and the immune system was shown by Rudolf Virchow more than 150 years ago (Adams et al., 2015). Interest in immune system activation as a therapeutic approach for treating cancer began in the late 19th century when William Coley injected heat-inactivated bacteria into the tumor mass, resulting in its size reduction. Although the failure to achieve desirable clinical outcomes with early immunotherapies such as interferon-gamma (IFN-γ) and interleukin (IL)-2 treatments, novel immunotherapies launched in the 21st century have achieved robust clinical results, establishing cancer immunotherapy as one of the foremost anchors of anticancer therapies (Lesterhuis et al., 2011; Jiang T. et al., 2016; Castro et al., 2018).

The effective eradication of cancer cells via the immune system involves several steps known as the cancer-immunity cycle, defined as a series of steps involving increased antitumor T-cell responses that are initiated upon recognition of the tumor-associated antigens (TAAs) captured from dying tumor cells by antigen-presenting cells (APCs) such as dendritic cells (DCs). Upon capturing TAA’s, DCs get activated, express CCR7, mature, and 1) migrate to draining lymph nodes, 2) present the captured antigens to naïve CD4^+^ and CD8^+^ T-cells via the major histocompatibility complex (MHC) class I and II molecules, 3) express T-cell costimulatory molecules, for example, CD40, CD80, and CD86, 4) secrete critical cytokines to regulate T-cell responses, 5) activate naïve CD8^+^ T-cells converting them into cytotoxic T-cells, which immigrate from lymphoid organs into the bloodstream and reach tissues and ultimately infiltrate the tumor. Activated cytotoxic T cells recognize the specific TAA (presented to them by DC’s) found on MHC class I (MHC-I) molecules of tumor cells and kill the tumor cells via secreting perforins and granzymes that result in the release of additional TAAs, which trigger the initiation of another cycle of cancer immunity (Chen and Mellman, 2013).

Cancer eradication through cytotoxic immune responses is evident; however, cancers can grow progressively, suggesting their ability to mask and not be recognized by the immune system as seen in carcinogen-induced mouse models. This mechanism prompted Schreiber and others to hypothesize the immunoediting concept to explain the progressive growth of otherwise immunogenic cancers (Shankaran et al., 2001; Dunn et al., 2004; Schreiber et al., 2011; Matsushita et al., 2012). The immunoediting process of human cancers can be related to neoepitope presentation. Non-silent point mutations that lead to antigenic neoepitopes (T-cell recognition) are lost more frequently in cancers than in silent point mutations, thus preventing T-cells from recognizing and identifying cancer cells (Rooney et al., 2015). This concept suggests that the ability of cancers to progress and grow could be impaired by loss of immunogenicity; however, this perception alone contradicts another evidence that T-cells are adequately activated to enhance their cancer recognition by the administration of immune-activating cytokines or immune checkpoints releases such as programmed cell death-1 (PD-1) or cytotoxic T-lymphocyte-associated antigen-4 (CTLA-4) that leads to robust tumor responses in patients and mice (Chambers et al., 2001; Pardoll, 2012). T cells are central infiltrates of the heterogeneous tumor microenvironment (TME), and their population consists of naïve, effector, memory, and regulatory T cells (Hashimoto et al., 2018). The antigen stimulation of T cell receptors (TCRs) initiates an intrinsic program that guides the differentiation of T cells into cytotoxic effectors capable of eradicating the antigen; however, these cells start dying gradually except for a small number of surviving memory T cells that provide long-term protection against the antigen (Chang et al., 2014). Chronic exposure of T cells to the same antigen leads to remarkable alterations, thus affecting their activation and differentiation and eventually causing T-cell exhaustion (Wherry, 2011; Schietinger and Greenberg, 2014). T effector cell exhaustion is highlighted by the loss of effector functions such as proliferation, cytotoxicity, metabolic and transcriptional molecule alterations, and immune checkpoint upregulation (Guo et al., 2018; Li H. et al., 2019). Different factors have been identified that play several roles in T-cell exhaustion; the intrinsic factors relate to transcription, epigenetic, and metabolic factors, whereas the extrinsic factors include extracellular and cytokine interactions that create the TME and the immunosuppressive network (Maimela et al., 2019; Zhang et al., 2020). Therefore, the use of engineered T-cells targeting specific cell-surface antigens is considered a great approach to ensure specificity and overcome the shortcomings of other available immunotherapies.

In this review, we present a comprehensive prospect of the developmental and experimental progress in the field of chimeric antigen receptor **(**CAR) T-cell therapy while relating to some aspects of adaptive immunity as the rationale behind the evolution of this cutting-edge technology. The significance of this review is the broad inclusiveness of current therapeutic applications of CAR T-cells in hematological malignancies, solid tumors, and human immunodeficiency virus (HIV) infection while focusing on some recently published results of pre-clinical and clinical trials, pointing out some drawbacks, and suggesting some modifications.

## 2 Adoptive Immune Therapy

Cancer immune therapy, which exploits the body’s immune system to combat cancer cells, can be classified into three categories: adoptive cell therapies (ACTs), tumor vaccines, and immune checkpoint inhibitors (ICIs). These therapies have proven beneficial in patients with advanced tumors, and some have reached complete remission (Li D. et al., 2019). ACT is mainly based on the concept that the immune system can control a patient’s cancer in the long-term and has been demonstrated by three independent approaches. The first approach involved tumor-infiltrating lymphocytes (TILs), which can be isolated from tumor lesions (e.g., melanoma) and expanded *in vitro*, followed by patient re-infusion, resulting in tumor regression and remission in a considerable number of patients. However, the downsides of the TILs approach included access limitations to the removable metastases or tumors, time-consuming preparation of T cells, and tumor-reactive T-cell clones were rarely found, which hindered the success of this strategy. The second approach involved T-cell receptor (TCR) engineering, where TCRs identified from TILs were virally transduced into peripheral blood T-cells, making them capable of inducing tumor regressions upon re-infusion into the patient. Unfortunately, this method was explicitly restricted because of its dependency on identifying MHC peptides expressed by tumors via their MHC complexes (Dudley et al., 2002; Zacharakis et al., 2018; Benmebarek et al., 2019). The third ACT approach is CAR-engineered T cells and is marked as the beginning of a new era in cancer therapy by providing a transformative approach to tumor exclusion and gained attention over the other two as it offered a series of innovative modifications (Kershaw et al., 2006; Lamers et al., 2011; Mikkilineni and Kochenderfer, 2017). CARs are synthetic receptors that have the specificity of a monoclonal antibody and a signaling domain capable of inducing a cascade of events in the CAR-engineered immune cells (e.g., T-lymphocytes) upon target engagement. Engineering immune cells to express CARs is achieved by transferring protein-coding sequences using viral vectors (e.g., Lentiviral or Retroviral). CAR T-cells display immunological characteristics similar to activated T cells such as generating an immune response against target cells and expanding within the patient ensuring long-term protection (Porter et al., 2011; Grupp et al., 2013; Heiblig et al., 2015).

## 3 Evolution of CAR-T Cells

**FIGURE 1:**
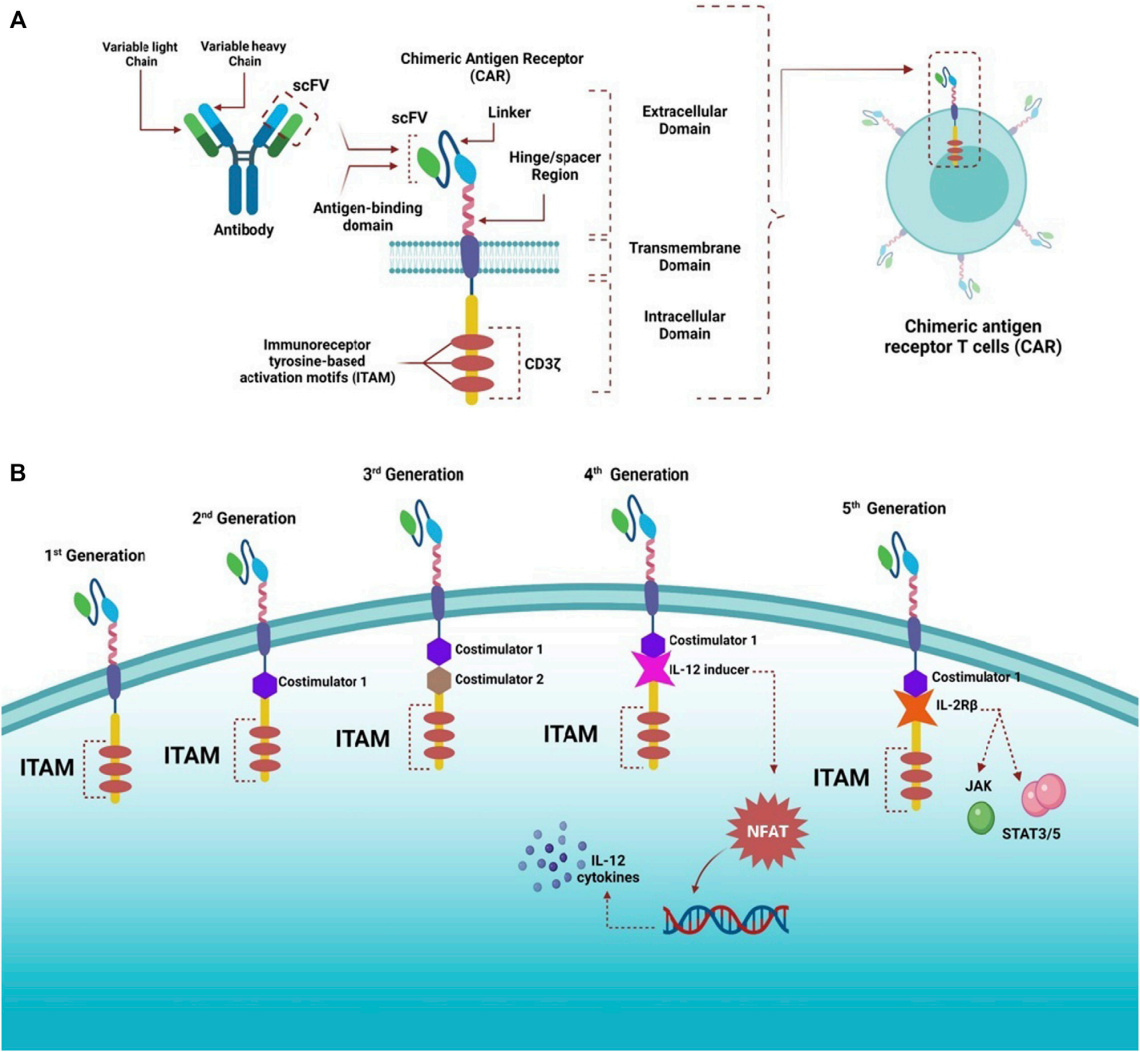
Structure of CARs and different generations. **(A)** Highlights the general structure of CARs; they have an extracellular domain containing scFV derived from antibody variable heavy and light chains, linker, and a hinge/spacer region. All the extracellular structures provide flexibility and improve the binding affinity of the antigen. A transmembrane domain helps anchor molecules to the T cells, and an intracellular domain containing ITAM motifs, responsible for transmitting activating and costimulatory signals to T cells, is also present. **(B)** CARs have witnessed rapid advancement since the first generation, which contained only ITAM (CD3ζ) motifs as the T cell stimulatory molecule within the intracellular domain. The second generation had one costimulatory molecule, whereas the third generation had two costimulatory molecules to improve cytotoxicity and robustness of CAR-T cells. The fourth generation was designed based on the second generation but was paired with cytokine expressors (e.g., IL-12) under the control of NFAT transcription factor; therefore, this generation is referred to as T cell redirected for universal cytokine-mediated killing (TRUCKs). The fifth generation was also based on the second generation with additional intracellular domains of cytokine receptors (e.g., IL-2Rβ) to activate JAK and STAT3/5, stimulate cell proliferation, and enhance its persistence.

Conventional T cells can distinguish between foreign peptide-MHCs (pMHCs) and the body’s pMHCs via their TCRs, which can trigger a small number of agonist pMHCs compared with thousands self-pMHCs (Sykulev et al., 1996; Irvine et al., 2002; Huang et al., 2013). Genetic insertion of CARs, in immune cells, particularly T-cells, redirects them to target a preferred antigen (Jackson et al., 2016). CARs are bioengineered receptors which specifically target a desired antigen; almost 30 years ago, the first CARs were generated and undergone multiple modifications since they contributed to their development and evolution (Kobold et al., 2015; Lim and June, 2017). The flexibility of CARs arises from their ability to recognize antigens in the absence of MHC presentation, which is the opposite of innate TCRs (Lim and June, 2017). Additionally, CARs have advanced properties compared with conventional T-cells, as they combine the antigen-binding ability of monoclonal antibodies with T-cell self-renewal and lytic capacity (Ramos and Dotti, 2011; Curran et al., 2012; Maher, 2012). Also, TCRs can recognize short peptide sequences, whereas CAR T-cells can recognize several tumor antigens in different forms, such as proteins, glycolipids, and carbohydrates (Abbott et al., 2020). CAR T-cell recognition and destruction of tumor cells occur in an independent-manner of MHCs; this promotes enhanced cell recognition undisturbed by the tumor’s ability to avoid MHC-restricted recognition of T-cells, such as the tumor’s ability to encourage defective antigen processing by downregulating human leukocyte antigen (HLA) class I molecules (Dotti et al., 2014). It is considered an advantage where MHC expression is suppressed or lost due to the immunosuppressive cancer microenvironment (Garrido et al., 2016). CARs have been proven effective in treating cancers, especially hematological tumors. The specificity of CARs in targeting cancers makes them an appealing alternative to standard cancer treatments such as chemotherapy and radiation (Sadelain et al., 2013). CARs consist of three major domains: 1) extracellular domain (ectodomain), which can be further divided into an antigen-recognition domain, a single peptide on the cell surface cleaved from the mature CAR cell (Goulart et al., 2017). The antigen-recognition domain is a single-chain fragment variant (scFV) chiefly comprising of heavy and variable light chain regions composed of an antigen-specific immunoglobin separated by a flexible linker and attached to the transmembrane domain by a spacer (hinge) responsible for the transmission of receptor-binding signals (Zhang et al., 2017). 2) transmembrane domain is essential for receptor stability and surface expression; it is a hydrophobic alpha helix that extends in the cell membrane (Ramos and Dotti, 2011; Zhang et al., 2017). 3) intracellular domain (endo-domain), which upon stimulation, clusters and undergoes conformational changes, thus enabling the recruitment and phosphorylation of downstream signaling proteins (Cantrell, 2002; Su and Vale, 2018). The intracellular domain classifies CARs into five generations: first has a single activation domain, a cytoplasmic domain mostly CD3 zeta (CD3ζ), and some studies used the gamma chain (γ) of the Fc receptors, the second generation has CD3ζ plus one costimulatory domain, obtained from costimulatory molecules such as 4-1BB or CD28 connected to an activator domain (CD3ζ/γ chain of Fc receptor) to enhance both cell proliferative and cytotoxic competences of CAR T cells (Finney et al., 1998; Hombach et al., 2001; Acuto and Michel, 2003). The third generation is similar to the second generation but has multiple costimulatory domains with CD3ζ, such as 4-1BB and CD28, CD134, and CD137 (Sadelain et al., 2013; Zhang et al., 2017; Guedan et al., 2019). The fourth generation CARs, known as T cells redirected for universal cytokine-mediated killing (TRUCKs), were engineered to release transgenic cytokine-like interleukin 12 (IL-12) upon CAR signaling in the tumor tissue to overcome TME immunosuppression and endorse robust therapeutic outcomes (Chmielewski et al., 2014; Chmielewski and Abken, 2015, 2020). IL-12 is responsible for the induction of IFN-γ, perforin, and granzymes in T-cells, and inhibits Treg proliferation (Kubin et al., 1994; Cao et al., 2009). Other cytokines studied in the fourth generation are IL-15 and IL-18 (Hurton et al., 2016). IL-15 belongs to the γ-chain family and holds important properties for T cell expansion and survival (Klebanoff et al., 2004). Additionally, IL-18 CAR T-cells treatment of large pancreatic and lung tumors exhibited changes in the immune cell landscape related to the tumor; a significant increase in the macrophages (CD206− M1) and NKs (NKG2D+) was observed besides a decrease in Tregs such as M2 macrophages suppressive CD103+ DCs, suggesting the ability of “IL-18 TRUCKs” to sensitize large tumor lesions for efficient immune destruction (Chmielewski and Abken, 2017).The fifth generation of CARs is currently being explored; it is mainly designed based on the second generation. However, it contains a truncated cytoplasmic receptor (IL-12) and a β-chain domain (IL-2Rβ truncated intracellular interleukin 2β chain receptor) along with the transcription factor STAT3/5 binding motif (Tokarew et al., 2019) (Figure 1).

The structure and design of CARs contribute to their signaling mechanisms, effector functions, efficacy, and toxicity. The ligand recognition and signaling of CARs are affected by both the single-chain variable fragment (scFv) and cytoplasmic domains; however, the transmembrane and spacer domains (non-signaling) affect the function of CARs (Jayaraman et al., 2020). Generally, CAR T-cells can specifically recognize cancer cells and lyse them (Maggs et al., 2021).

## 4 Clinical Preparation of CAR-T Cells

Despite various designs and tumor-specific scFVs, the manufacturing process of CAR-T cells remains constant (Wang and Rivière, 2016). In general, the personalized clinical production of CAR-T cells encompasses several steps followed by quality control testing through the entire process (Levine, 2015). The first step is collecting leukocytes from the patient (autologous) or the donor (allogeneic) from the peripheral blood via leukapheresis, in which only the leukocytes are extracted, and the rest of the blood products are returned to circulation (Brown and Adusumilli, 2016; Zhang et al., 2017). Second, T cells are augmented, separated, and washed with leukapheresis buffer (Zhang et al., 2017; Gomes-Silva and Ramos, 2018). Third, at the CD4/CD8 composition level, the T-cell subsets are separated using specific antibody-coated bead conjugates or markers. The isolated cells are then cultured and activated by purified allogeneic or autologous APCs or by introducing beads coated with anti-CD3 or anti-CD28 monoclonal antibodies (or both along with feeder cells and interleukins) (Guedan et al., 2019). IL-2 is the most common growth factor used to induce the rapid growth of T cells (Wang and Rivière, 2016; Guedan et al., 2019). Recently, a study reported that a cytokine cocktail of IL-2, IL-7, and IL-15 induced better expansion of CD4 and CD8 CAR-T cells (Coppola et al., 2020).

Fourth, different methods have been considered to enable nucleic acid delivery to the obtained T cells. Usually, a foreign gene material (RNA or DNA) delivery into human cells can be accomplished using viral or non-viral vectors. Viral vectors are preferable for basic and clinical research because viruses have diverse expression characteristics, spend a fraction of time to reach clinically desired numbers of cultured T cells, and possess high transfer competency (Zhang et al., 2017; Gomes-Silva and Ramos, 2018). Viral vectors are used to encode CARs; with their reverse transcription potential, vectors convert RNA into permanently integrated DNA in the genome of the obtained T cells. These viral vectors include retroviruses, lentivirus, adenovirus, and adeno-associated virus. The most popular ones are genetically engineered retroviruses, more frequently used than gamma retroviral vectors. During the activation period, viral vectors are washed out of the culture by dilution and medium exchange (McGarrity et al., 2013; Zhang et al., 2017).

However, viral vectors present a possible safety hazard. The limitations of the viral vectors include tumorigenesis and toxicity caused by the insertion mutation used to generate immune reactions, and the limited carrier capacity and achieved titers are not sufficient (Wang et al., 2008). Therefore, to overcome the shortcomings of viral vectors, other methods such as mRNA transfection and non-viral vectors were used in the production of CAR-T cells. The most common were transposon-based non-viral vectors, facilitating safe and consistent DNA transfer into CAR T-cells. The sleeping beauty (SB) transposon system is the currently used substitute for viral-based vectors. It has been used to prepare CD19^+^ CAR T-cells with antitumor properties *in vivo* and *in vitro* (Singh et al., 2015; Chicaybam et al., 2019). In 2014, an optimized protocol (GMP-compliant) was suggested to utilize the production of modified CAR T-cells by electroporation with CAR-encoding RNA, which helps in overcoming several drawbacks of classic viral transfection such as viral contamination, low time-efficiency, higher resource consumption, and off-target effects (Krug et al., 2014). In 2019, the optimized protocol was used in producing genetically modified CAR T-cells against melanomas; the CAR T-cells were electroporated and expanded with mRNA that encoded CAR targeting CSPG4, a surface protein highly expressed in most melanomas. The results showed that a high dosage of modified CAR T-cells could lyse 80% of melanoma cells after 20 h; the authors suggested a future expansion of their study to a full clinical trial (Wiesinger et al., 2019).

**FIGURE 2:**
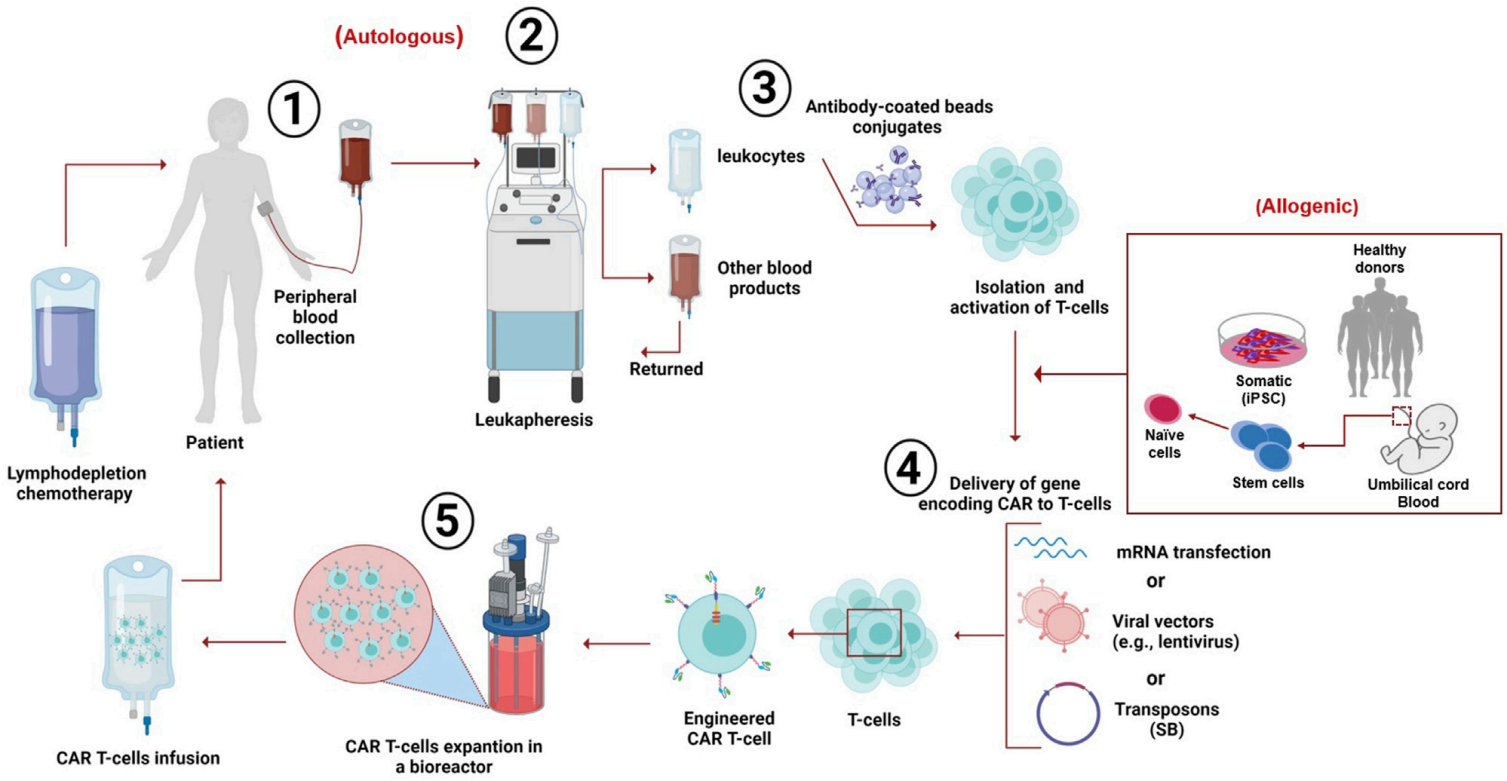
Clinical production of CAR T-cells. The peripheral blood is withdrawn from the patient (autologous) or it can be obtained from the peripheral blood of a healthy donor (mononuclear cells), induced pluripotent stem cells (iPSC), or umbilical cord blood (allogeneic). The targeted T-cells are obtained by leukapheresis. Then, the T cells are separated and purified from other leukocytes using anti-CD3/CD28-coated beads; this process is followed by activation of the cells. Then, the genetic material encoding chimeric receptors is introduced into the T-cells via several known methods (such as mRNA transfection), viral vectors (e.g., lentivirus), or sleeping beauty (SB) transposons. The engineered T-cells expressing CARs are then expanded in a bioreactor. The patient receives chemotherapy for decreasing white cells blood count; after 48–96 h, the CAR T-cells are reinfused into the patient, followed by close monitoring for a few days to observe any adverse effects.

This approach has several advantages, such as improved integration of the transduced genetic material due to its low promoter activity (Yant et al., 2000), fewer epigenomic changes at the integration site, and reasonably low manufacturing costs (Izsvák et al., 2010). The only limitation in this approach is the low rate of transgenic material; however, it was considerably enhanced (Geurts et al., 2003). Nevertheless, the concerns remain; for instance, transient mRNA transfection requires several rounds of infusion, the possibility of mutagenesis, and SB transposon remobilization (Beatty et al., 2014). The fifth step is CAR-T cell expansion using bioreactors, which help cells divide and express CARs on the cell surface (Harrison et al., 2019). Finally, when the cells reach the clinically required volume, they are reinfused into the patient as a therapeutic agent. The infusion occurs 48–96 h after lymphodepletion chemotherapy to make room for the infused CAR-T cells (Turtle et al., 2016). The patient is then kept under observation for possible adverse effects within the first few days of infusion. The process lasts around 3 weeks, where cell preparation is the most time-consuming phase of treatment (Zhao and Cao, 2019) (Figure 2).

Interestingly, lymphodepletion chemotherapy is a crucial step before CAR T-cells infusion as it reduces endogenous lymphocyte numbers, thus increasing hemostatic cytokine availability promoting infused cells survival (Liang et al., 2020). Administration of T-cells to lymphodepleted patients has shown superior anti-tumor properties compared to lymphoreplete patients (Bechman and Maher, 2021). There have been several benefits to the lymphodepletion regimens, such as the non-myeloablative chemotherapeutic approach; this regimen results in the removal of endogenous lymphocytes that act as “cytokines sinks,” which facilitate the accessibility of the infused T-cells to hemostatic cytokines like IL-15, IL-7, and IL-2, which stimulate JAK-STAT-mediated expansion (Gattinoni et al., 2005; Neelapu, 2019). In a lymphodepleted host, the memory cells proliferate in an antigen-dependent manner, unlike naïve T cells homeostatic expansion, which occurs in an MHC-dependent manner (Gattinoni et al., 2005; Klebanoff et al., 2005). It has been reported that lymphodepletion decreases immunosuppressive cells, such as myeloid-derived suppressor cells (MDSCs) and regulatory T cells (Tregs), while enhancing the APC cells’ functionality and availability (Bechman and Maher, 2021). Immunosuppressive networks are negatively affected by lymphodepleting agents such as tryptophan metabolizing enzyme and indoleamine dioxygenase (IDO) (Hanafi et al., 2014; Ninomiya et al., 2015). Lymphodepletion also exerts certain positive effects on the microbiome. It enhances the translocation of microbes from the gastrointestinal tract, which lead to immunostimulatory impacts through Toll-like receptor ligation, resulting in an augmented release of IL-1β (Viaud et al., 2013; Lee et al., 2019). According to imaging studies, post lymphodepletion, the tumor-trafficking properties of adoptively infused cytotoxic T-cells were enhanced (Pittet et al., 2007). In a clinical trial (NCT03939026), which evaluates the safety and efficacy of certain lymphodepletion regimens, the phase I results suggest that fludarabine as a component in the lymphodepletion regimen is critical and contributes to the efficacy of the procedure. Moreover, using a combination of fludarabine and cyclophosphamide (Flu/Cy) regimen is beneficial in multiple tumors; however, this combination is required for optimization in certain types of cancer and attenuation of the exerted toxicities of these agents. Although the benefits of lymphodepletion are undeniable, there have been certain limitations, such as the short-lived span of lymphodepletion and the consequent immune restoration phase accompanied by a compensatory overshoot of both MDSCs and Tregs as indicated by preclinical and clinical studies (Bechman and Maher, 2021).

**FIGURE 3:**
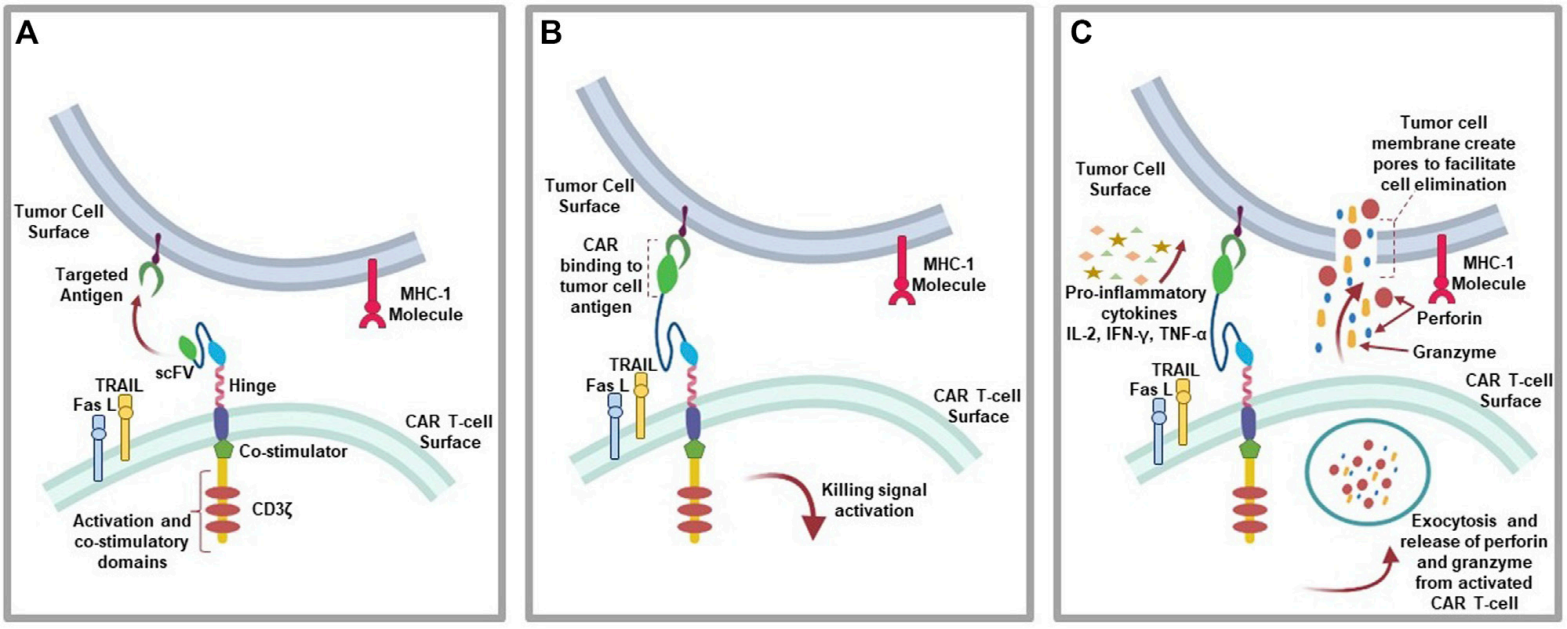
CAR T-cell action: **(A)** CAR T-cells recognition of targeted antigen. **(B)** Chimeric antigen receptor binding to tumor-antigen. **(C)** Initiation of the antitumor (cytolytic) effects where the activated T-cells downstream the killing signaling by secreting granzymes and perforins, pro-inflammatory cytokines due to immune cell invasion, as well as initiating the expression of TRAIL and FasL pathways.

The mechanism of action of CAR T-cell involve the binding of CARs to a targeted antigen present on tumor cell surface via scFV recognition domain, which elicit anti-tumoral effects through the secretion of inflammatory cytokines (e.g., IL-2, IFN-γ, and TNF-α), cytolytic effector function via perforin and granzyme (Benmebarek et al., 2019), TNF-related apoptosis-inducing ligand (TRAIL), which binds to death receptors (e.g., DR4 and DR5) on tumor cells cell surface to activate graft-versus tumor effect (donor T-cells) (Watanabe et al., 2021). Also, tumor cell apoptosis can be initiated via the activation of caspase 8 and the formation of death-inducing signaling complex (DISC) leading to cell death mediated by mature caspase 3 subsequent cleavage of over 500 cellular substrate as a result of Fas and Fas ligand (FasL) pathway activation (Waring and Müllbacher, 1999; Nagata and Tanaka, 2017) (Figure 3).

## 5 Clinical Applications of CAR-T Cells

### 5.1 Hematological Malignancies

#### 5.1.1 Acute Lymphoblastic Leukemia

CAR-T cells are primarily used in hematological malignancies such as Acute lymphoblastic leukemia (ALL), characterized by a rapid proliferation of naïve cells in the bone marrow. CAR-T cells showed efficacy in treating ALL, especially the engineered T cells against CD19, as CD19 is a highly expressed biomarker of the B-cell lineage, responsible for B-cell malignancy of ALL. CD19 is a transmembrane glycoprotein involved in B-cell activation and is expressed throughout the developmental stages of B cells (Wang et al., 2012). Another potential target in B-ALL is the light chain of immunoglobulin CD20 (Gill et al., 2016; Jain and O’Brien, 2016). Conversely, T-cell malignancy of ALL (T-ALL) showed limited efficacy when the engineered CAR T-cells targeted CD19; therefore, another target (anti-CD5) showed effective elimination of a specific T-cell line expressing CD5 (Mamonkin et al., 2015). Anti-CD4 CAR-T cells are potential targets showing promising results against T-cell lymphoma (CD4 positive) models *in vivo* and *in vitro* (Pinz et al., 2016)*.* Clinical trials evaluating multitargeted CAR-T cell therapy against ALL, such as single engineered CAR-T cells targeting both CD19 and CD22 and a combination of CAR-T cells with anti-CD19 and anti-CD20 to target each antigen independently, have yielded encouraging results (Huang et al., 2018). KYMRIAH ™ (tisagenlecleucel), which is a second generation CAR-T cell product (4-1BB costimulatory domain) directed against CD19 antigen, was approved by the Food and Drug Administration (FDA) for ALL in 2017 based on multicenter clinical studies, which established an overall remission rate of 81% in children and young adults with relapsed/refractory acute B-cell acute lymphoblastic leukemia (r/r B-cell ALL), and a best overall response rate of 52% in adults with relapsed/refractory diffuse large B-cell lymphoma (r/r DLBCL) (Thudium Mueller et al., 2021).

#### 5.1.2 Acute Myeloid Leukemia

Acute myeloid leukemia (AML) results from genetic alterations in precursor cells that affect the growth and differentiation of hematopoietic cells, resulting in the accumulation of immature myeloid cells in the bone marrow and peripheral blood. These cells are incapable of turning into mature hematopoietic cells. CAR-T cell therapy in AML did not show the same success as seen in ALL, and the target of CAR T-cells in AML was CD123 and CD33; the latter was used in treating a patient and showed a significant reduction in tumor volume in the bone marrow; however, 9 weeks post-infusion, the patient experienced a relapse (Wang et al., 2015). Furthermore, the use of anti-CD123 CAR-T cells as a potential treatment of AML showed inadequate potency in “on-target-off-tumor” since CD123 is also expressed in normal tissues (e.g., endothelial tissue) and monocytes in relatively low levels compared with AML (Tettamanti et al., 2014). Therefore, other antigens have been investigated as new targets, including Lewis-Y (LeY) and CLEC12A antigens, and anti-LeY CART-cells were used in patients who eventually developed disease progression.

In contrast, the CD33was used as anti-CLEC12A-CD33 CAR T-cells, showed complete remission in a 44-year-old female patient with refractory AML (Ritchie et al., 2013; Morsink et al., 2019). In AML the application of CAR T cell therapy is limited by the absence of an AML-specific antigen. AML cells can express several cell surface antigens such as CD34, CD33, CD123, and many more. moreover, these antigens are also expressed by healthy Hematopoietic stem and progenitor cells (HSPCs) and their lymphoid and myeloid progenitors (Cummins and Gill, 2019). However, CAR-T cells are unable to distinguish between malignant and normal cells, unlike CD19 CAR T-cells, their complete elimination of the normal and malignant B cells resulting in B cell aplasia is considered clinically benign and manageable by intravenous immunoglobulin infusion; however, this is not the same in targeting myeloid antigens shared with normal myeloid progenitor as their elimination could be fatal due to bleeding complications and neutropenic infections (Mardiana and Gill, 2020). The aggressiveness of this disease and its ability to develop resistance against treatments requires substantial efforts to achieve remission. CAR T-cell therapy is a promising technology; however, the lack of leukemia-specific cell surface antigens could present a problem in designing CAR T-cells against AML (Mardiana and Gill, 2020). HSPCs frequently share antigens with AML. CAR T-cells expansion is threatened by AML blasts or prior exposure to chemotherapy that damages T cells. In addition, the AML’s ability to evade the immune system by inducing various immunosuppressive mechanisms makes it challenging to achieve desired outcomes (Mardiana and Gill, 2020).

#### 5.1.3 Chronic Lymphocytic Leukemia

Chronic lymphocytic leukemia (CLL) results in excessive mature lymphocytes in the blood, bone marrow, and lymphoid tissue (Kipps et al., 2017). The use of CD19 as a target in the case of CLL by producing anti-CD19 CAR-T cells has shown remarkable results in patients with complete remission and minimal residual disease, and anti-CD19-CD28ζ CAR-T cells have shown promising results, according to the data from the National Cancer Institute (Porter et al., 2011, 2015; Kochenderfer et al., 2015). Pharmacokinetics plays an essential role in enhancing the outcomes and safety of CAR-T cell treatments, especially when it comes to the individual persistence of treatments, as it is considered the main goal in achieving the desired long-term antitumor effects (Norelli et al., 2016). In a recent study (NCT01747486), 42 patients (18 years and above) with CLL were treated with autologous CD19 CAR T-cells and 38 patients were infused with anti-CD19 CAR T-cells. Twenty-eight patients randomly received a low dose of (5 × 10^7^) and high dose of (5 × 10^8^), and 24 were evaluable for response assessment. After a short time, ten patients revived the high dose while eight were evaluable for response assessment. Follow-up ranged from 2 to 75 months; results showed that higher doses effectively induce a complete remission (CR) without excessive toxicities (Frey et al., 2020). One of the inevitable issues in CLL treatment is the antigen-negative relapse that has been threatening CAR T-cell therapy’s success in CLL patients (Mancikova and Smida, 2021). The proportion of remissions in patients with CCL post CAR T-cell therapy remains the lowest compared to the spectrum of B-cell tumor patients. Current data are crucial for utilizing the clinical effects, and ibrutinib administration and partial reversing of the exhausted phenotype of CAR T-cells in CLL patient seem substantially promising. The genetic modification (insertion) of transgenic vector in the recipient T cells with systems such as CRISPR/Cas9 may contribute to treatment efficacy. The low quality of non-functional CAR T-cells derived from treated CLL patients could be improved using allogeneic CAR T-cells. The antigen-negative relapse could be alleviated using bispecific CARs targeting two antigens presented on the tumor cell surface. Suitable biomarkers must be identified and used as targets to design treatment and avoid infusion failure (Mancikova and Smida, 2021).

#### 5.1.4 Non-Hodgkin’s Lymphoma

Non-Hodgkin’s lymphoma (NHL) consists of a group of neoplasms with various degrees of malignancy occurring in lymphocytes, lymphoid tissue, and histocytes at any stage of their development. These heterogeneous lymphoproliferative malignancies have a greater chance of dissemination to extranodal sites and are less predictable than Hodgkin’s lymphomas (HL) (Singh et al., 2020). Anti-CD19 CAR-T cells have shown remarkable results in treating chemo-resistant lymphomas. Patients with refractory diffuse large B-cell lymphoma (DLBCL) had complete remission for more than 2 years (Neelapu et al., 2017a; Schuster et al., 2017; Tiberghien et al., 2017). CD22 is expressed in progenitor and differentiated B cells and is highly expressed in B-cell lymphomas and leukemia. Anti-CD22 showed promising results in four out of nine patients with a negative minimal residual disease and complete remission (Fry et al., 2015). Anti-CD20 and anti-CD23 CAR-T cells have also been used to treat NHL (Till et al., 2012; Zou et al., 2018). In 2017, the FDA approved YESCARTA (axicabtagene ciloleucel), which is a CD19 directed second generation CAR-T (CD28 co-stimulatory domain) cell product for the treatment of NHL.

#### 5.1.5 Hodgkin’s Lymphoma

Hodgkin’s lymphoma (HL) is a common lymphoma derived from B cells. Hodgkin and Reed-Sternberg cells are rarely found in the tissues derived from mature B cells that lose their phenotype and co-express unusual hematopoietic cell markers (Küppers et al., 2012). HL cells highly express CD30; therefore, it was considered an ultimate target by engineered CAR-T cells, and clinical trials showed encouraging results where patients diagnosed with HL exhibited complete remission after anti-CD30 CAR-T cell therapy, wherein other patients either developed stable disease or relapse; however, the observations of anti-CD30 CAR-T cells did not show any toxicities or adverse events (Ramos et al., 2017; Wang et al., 2017).

#### 5.1.6 Multiple Myeloma

Multiple myeloma (MM) is also a B-cell malignancy of long-lived plasma cells, which play a significant part in the immune defense system by producing antigen-specific immunoglobulins; in the case of malignancy, these cells excessively produce a specific immunoglobulin (containing two heavy chains and two light chains) and additional light chains, which can be detected in the blood. They are used to diagnose and monitor MM (Bird and Boyd, 2019). Disease management was compromised because of the unavailability of an ideal target. Syndecan 1 (CD138) was the target for the treatment of MM. This surface protein was expressed on both plasma cells and normal cells (epithelial), causing “on-target-off-tumor” toxicity. Nonetheless, Chinese clinical trials using CD138 as a target achieved stable disease in 4/5 patients (Heffner et al., 2012). Another target is the B-cell maturation antigen (BCMA), which is thought to be involved in all stages of B-cell differentiation and maturation and is highly expressed in myeloma cells; therefore, it is considered a better target in CAR-T cell therapy (Ali et al., 2016). A phase 1 clinical trial showed preliminary results regarding BCMA (anti-CD269) CAR-T cell therapy; one patient achieved complete remission for more than 3 months, whereas another patient showed an outstanding partial response to therapy. Additionally, a correlation was established between high treatment efficacy and higher doses. However, the higher the dosage, the more adverse events were seen, such as cytokine release syndrome, regardless of the use of BCMA or CD138 in therapy (Yang X. et al., 2019). The expression levels of CD19 in the plasma cells were low, but they were observed to be slightly higher in malignant cells and showed remission in a 43-year-old patient using CTL019 cells and CD19 as a target in MM. Cytokine release syndrome did not develop, and following several days of infusion, CTL019 cells were detected in the bone marrow and blood (Garfall et al., 2015). BCMA CAR T-cells were designed with signaling domain (CD3ζ) and CD28 (costimulatory domain) in a study (NCT02215967) conducted with 24 patients with MM; the cytotoxicity observed was minor post an infusion of a minimum dose (0.3–3.0 × 10^6^ cells/kg). The objective response rate (ORR) was 20%. The anti-tumor function with 81% ORR, while severe cytokine release syndrome (CRS) was reported in higher dosage of CAR T-cells (9 × 10^6^ cells/kg) (Brudno et al., 2018). Bispecific CAR T-cell (LCAR-B38M) was designed to target VHH1 and VHH2 epitopes of BCMA in a multicenter study (NCT03090659) on patients with MM. The findings included 88% ORR and 68% CR. The adverse events included leukopenia, thrombocytopenia, CRS, and pyrexia (Zhao W.-H. et al., 2018). In 2021, the FDA approved ABECMA (idecabtagene vicleucel) for MM. ABCEMA is a second generation CAR-T cell product directed against the BCMA tumor antigen.

### 5.2 Solid Tumors

#### 5.2.1 Renal Cancer

Renal cancer (RCC) is one of the most diagnosed cancers in both men and women worldwide. RCC development is associated with several factors, including chronic kidney disease, smoking, hypertension, and obesity (Rossi et al., 2018; Capitanio et al., 2019). For many years, surgical intervention was the most effective treatment for RCC, known for its chemoresistance. Later, other treatments such as cytokine and tyrosine kinase inhibitors (TKIs) were approved, and when RCC showed possible immunological sensitivity, other immunotherapies were approved as well (Schepisi et al., 2020). CAR-T cell therapy of RCC targets carboxy-anhydrase-IX (CA-IX) as an antigen, which participates in the catalysis of carbon dioxide hydration (Bagley and O’Rourke, 2020; Bagley and O’Rourke, 2020) and is considered a critical antigen in RCC; however, it is also found in other normal tissues of gastric mucosa epithelium, small intestine epithelium, duodenum, and the biliary tree where it is expressed moderately (Yeku et al., 2017). The expression of CA-IX can be induced under hypoxic conditions in various tissues (Tafreshi et al., 2014). The first generation of CA-IX/CART-cells toward RCC was associated with high cytokine secretion due to cytotoxicity (Li et al., 2018).

#### 5.2.2 Ovarian Cancer

Novel therapeutics are constantly required in Ovarian cancer (OC) as it is known for its high recurrence levels post-surgery and multi-agent chemotherapies. CAR-T cells are a novel therapy. In the context of ovarian cancer, they target tumor-associated glycoprotein 72 (TAG72); humanized TAG72-specific CAR-T cells exhibited cytokine production and cytotoxic activity in OC. In contrast, it also showed proliferation reduction and increased mouse viability in mouse models (Murad et al., 2018). Another target was mucin 16 (MUC16), which causes OC progression depletion after intraperitoneal and intravenous injection in mouse models, making it one of the potential targets, and an *in-vitro* study using Her-2 CAR-T cells on human OC cell line (SKOV3) expressing Her-2/neu reported growth suppression potential (Chekmasova et al., 2010). The antigen mesothelin was targeted by anti-Meso CAR-T cells, which inhibited proliferation and increased mouse viability. Additionally, 5T4-specific CAR-T cells and FRα-specific CAR-T cells exhibited inhibitory effects against OC cellular growth and progression (Zuo et al., 2017; Owens et al., 2018). In the dual design of CAR-T cells targeting both CD19 and mesothelin (MSLN-CAR NK-92) cells using lentivirus gene transfer, the MSLN-CAR molecules were highly expressed on the surface of NK-92, which led to the killing of MSLN^+^ OC cells such as SKOV3 and OVCAR3 *in vitro* (Cao et al., 2020).

#### 5.2.3 Lung Cancer

Lung cancer is one of the most diagnosed cancers worldwide and is considered one of the leading causes of death. Several antigens have been targeted to treat this cancer, including epidermal growth factor receptor (EGFR), which is highly expressed in the epithelium and epithelium-derived tissues compared with normal lung tissues. Because the receptor provides significant affinity for binding sites in lung carcinomas, it is one of the most therapeutic targets of CAR-T cells. Second-generation EGFR-CAR-T cells with CD137 co-stimulatory domain showed feasibility and safety in treating refractory/relapsed non-small cell lung cancer (Feng et al., 2016). Another candidate target was HER2, as it exhibited good therapeutic outcomes in refractory/recurrent HER2^+^ sarcomas without any respiratory distress syndrome (RDS) signs. However, RDS was observed 15 min after cell infusion in one patient diagnosed with metastatic colon cancer to the lung and liver, plausibly because of an autoimmune reaction. Generally, the safety and efficacy of this anti-HER2 CAR-T cell in lung cancer depends on the levels of HER1 in patients and might be compromised because of RDS (Morgan et al., 2010).

Further antigens were considered, including MSLN, since it is expressed in 69% of lung adenocarcinoma (1/5 patients) and not in normal lung tissues and reduced tumor burden in mouse models (Carpenito et al., 2009; Kachala et al., 2014). The NSCLCs were found to overexpress transmembrane glycoprotein MUC1 and Prostate Stem Cell Antigen (PSCA), a glycosylphosphatidylinositol (GPI)-anchored cell surface antigen; therefore, they were preferred to be used in combination as potential targets for MUC1-CAR-T cells and anti-PSCA-CAR-T cells, which showed excellent efficacy compared with using a single antigen (Wei et al., 2017). Carcinoembryonic antigen (CEA) is overexpressed in nearly 70% of NSCLCs (Berinstein, 2002); however, patients who received anti-CEA CAR T-cell treatment had transient acute respiratory toxicity, possibly because of the expression of CEACAM5 on lung epithelial cells (Thistlethwaite et al., 2017). The tyrosine kinase-like orphan receptor 1 (ROR1) was used as a target; however, toxicity concerns are growing since it was also expressed in normal tissue. Therefore, to overcome this issue, selectivity of the target was improved by engineering CAR T-cells with synthetic Notch (synNotch) receptors specific for EpCAM or B7-Homolog 3 (B7-H3), a member of the B7 family of immune checkpoint molecules, which is expressed on ROR1+ tumor cells but not on ROR1^+^ stromal cells, resulting in the regression of tumor cells without causing toxicity (Srivastava et al., 2019). The costimulatory role of CD80/CD86 makes it a suitable target for immune intervention, and upon binding to CTLA4 (CTLA4-CD80/CD86), T cells are downregulated via various mechanisms. In several NSCLC cells, the mRNA expression of CD80/CD86 was detected in normal tissues, risking autoimmunity reactions; hence, new strategies are encouraged to overcome this risk by using CD80/CD86 CAR-T cells and enhancing its selectivity (Wroblewski et al., 2001; Egen et al., 2002).

#### 5.2.4 Breast Cancer

Breast cancer (BC) is one of the leading causes of death in women, wherein 1.5 million women are diagnosed with BC worldwide each year. BC is diagnosed during routine screening or incidentally, and it could reach the lymph nodes and metastasize to other organs such as the brain (Sun et al., 2017; Seely and Alhassan, 2018). One of the most attractive targets for CAR T-cell therapy is triple-negative breast cancer (TNBC). This type of breast cancer lacks estrogen (ER), progesterone, and epidermal growth factor (EGFR) receptors (Harrer et al., 2019). The targeted receptors for CAR T-cell treatment include folate receptor alpha (FRα); as a result, the anti- FRα CAR T-cells killed *in vitro* TNBC cells. This antitumor activity correlates with the FRα antigen levels in the cells (Song et al., 2016). The MUC1 antigen is associated with different tumor invasiveness and metastatic behavior, including breast cancer, making it a potential treatment target (Zhou et al., 2019). Integrin αvβ3 is another tumor antigen expressed in different tumors, including BC tumors, and stimulates tumor cell survival and metastasis (Felding-Habermann et al., 2001). Tyrosine-protein kinase Met (c-Met) is a cell surface molecule expressed in almost 50% of breast tumors. After an intratumoral injection of c-Met CAR mRNA, the tumors were excised and analyzed via intratumoral injection immunohistochemistry, revealing inflammatory and necrotic responses (Tchou et al., 2012; Zhao et al., 2017). The ROR antigen was also used as a CAR T-cell target in BC, eliminating multiple layers of tumor cells deep in the tumor tissues above and beneath the basement membrane (Wallstabe et al., 2019). Recent clinical trials have targeted several antigens against BC, including HER2, MUCI, CEA, CD70, CD133, ROR1, and NKG2D ligands (Williams et al., 2017). The cell surface antigen mesothelin was found to be overexpressed in 67% of TNBC samples and is considered a potential target because of its involvement in the activation of intracellular pathways including MAPK, NFкB, and PI3K, resulting in tumor cell proliferation and resistance to apoptosis (Morello et al., 2016; Tchou et al., 2017). CSPG4 is a tumor glycoprotein found in 72.7% of TNBC lesions and believed to be associated with tumor cell survival and recurrence; it was primarily detected in TNBC stem cells responsible for resistance and relapse. Using anti-CSPG4 CAR T-cells in TNBC metastasis and progression can also be diminished; it can attack more than one target, including stromal cells, primary TNBC cells, and cancer-associated fibroblasts, which are considered to be crucial for maintaining the TME (Wang et al., 2010; Cooney et al., 2011; Harrer et al., 2019). Disialoganglioside GD2 is a BC stem cell antigen expressed in 35.5% of metastatic TNBC and is considered an immunotherapeutic target, and anti-GD2 CAR T-cells have been reported to show cytolytic activity in GD2^+^ cell lines (Seitz et al., 2020; Xia et al., 2020). The TEM8 marker was found to be overexpressed in the vasculature of solid tumors. When anti-TEM8 CAR T-cells were used in the TNBC mouse model, explicit control of the tumor growth was observed without exhibiting any toxicity. On the other hand, in healthy mouse models, cytotoxic effects were observed, which might be due to the retroviral vectors used that might have affected the abundance of CAR T-cells (Risma and Jordan, 2012b; Chaudhary et al., 2012; Byrd et al., 2018). Another intriguing target is the human endogenous retrovirus family K (HERV-K) antigen, highly expressed in basal BC cells, similar to TNBC. Importantly, it is absent in nearly all normal human tissues. The anti-K CAR T-cells experimented with *in-vivo* BC mouse models showed slow tumor growth. The MDA-MB-231 cell line showed great lysis post-exposure to anti-K CAR T-cells prepared from cells obtained from patients with BC (Zhao et al., 2011; Wang-Johanning et al., 2012; Krishnamurthy et al., 2015; Zhou et al., 2015, 2016; Johanning et al., 2017).

#### 5.2.5 Prostate Cancer

The second most frequently diagnosed malignancy in men is prostate cancer (PrC) and the fifth leading cause of death worldwide. According to GLOBOCAN 2018, the number of newly reported diagnoses in 2018 reached 1,276,106 cases worldwide, with a higher incidence in developed countries (Rawla, 2019). Prostate-specific membrane antigen (PSMA) has been used as a target by CAR T-cells in studies (*in vivo* and *in vitro*) and causes the proliferation and differentiation of PSMA^+^ cells (Maher et al., 2002; Gade et al., 2005). In mouse models of metastatic PrC, diabetes, and severe combined immunodeficiency, the use of PSMA CAR T-cells eradicated metastatic PrC cells. The second generation CAR T-cells (containing co-stimulator CD28) offer a novel immune-targeted approach for metastatic PrC since it showed a better eradication effect than the previous generation (Ma et al., 2014; Zuccolotto et al., 2014). The anti-PSMA CAR T-cell dosage and protocols for metastatic PrC patients is being investigated in phase 1 clinical trials, in addition to the possible use of dual-targeted CAR T-cells targeting PSMA and transforming growth factor-β (TGFβ) and their safety in another phase 1 clinical trial (Slovin et al., 2013; Kloss et al., 2018). The prostate stem cell antigen (PSCA) is also an attractive target for CAR T-cell therapy; the first generation of CAR T-cells with the scFV of 7F5 antibodies exhibits antitumor effects in mice. In another study that used the 4-1BB co-stimulator, the activation of T cells was better than that by the CD28 co-stimulator (Hillerdal et al., 2014; Priceman et al., 2018). As a potential strategy, combined CAR T-cell therapy uses low-affinity PSCA CAR T-cells and high-affinity PSMA CAR T-cells to eliminate double-positive CAR T-cells in PrC (Feldmann et al., 2017). A different approach is to use diabodies (bispecific antibodies; BITEs) that simultaneously bind to specific T-cell receptor-associated molecules on the T-cell surface (e.g., CD3ε) and to a tumor-specific antigen expressed on the cancer cell surface (e.g., CD19; PSMA). The simultaneous engagement of BITEs with both CD3 and the specific antigen resulted in tumor cell lysis via the activation of cytotoxic T-cells. BITEs have also been reported to be overexpressed in tumor tissues compared to normal ones (Stone et al., 2012; Stieglmaier et al., 2015; Yang et al., 2016). These novel antibodies were evaluated in combating cells by targeting PSMA (Baum et al., 2012; Friedrich et al., 2012; Feldmann et al., 2017). In animal models, these novel antibodies failed to block the proliferative activity of cancer; they only caused delayed tumor growth, which suggests that the use of diabodies as a single treatment would not achieve a sturdy cellular memory response (Hillerdal and Essand, 2015). However, in murine xenograft PrC models, the humanized bispecific antibody MOR209/ES414 caused tumor growth inhibition and improved survival. PSMA expression was reduced only in transferred and adaptive human T cells. In a recent study on xenograft models, BITE targets CD3 in T cells and PSMA in PrC cells. The results revealed their antitumor potential (Hernandez-Hoyos et al., 2016; Bailis et al., 2019). An additional target of PrC is the epithelial cell adhesion molecule (EpCAM; also known as CD326), a known stem cell antigen present in several tumors, including PrC (Gires et al., 2009; Ni et al., 2012). Recently in Europe, EpCAM-CD3 was approved for the treatment of malignant ascites. Using it as a TAA, it was developed to produce anti-EpCAM CAR T-cells capable of combating PC3M cells overexpressing EpCAM, thereby extending the survival of under-expressing EpCAM PC3 cells. However, further investigation of its efficacy in metastatic PrC is needed (Deng et al., 2015).

#### 5.2.6 Liver Cancer

Liver cancer is a global health burden, with an estimated >1 million cases by 2025. The most frequently diagnosed type of liver cancer is hepatocellular cancer (HCC), contributing ∼90% of all diagnosed cases. Many risk factors play a role in the progression of various diseases, such as hepatitis B and C infection, non-alcoholic steatohepatitis associated with diabetes mellitus, or metabolic syndrome (Llovet et al., 2021). The glypican-3 (GPC3) cell surface has been targeted in CAR T-cell therapy against the HCC xenograft mouse model and proved effective (Gao et al., 2014; Jiang Z. et al., 2016). Other targets are being investigated, including MUC 1, CEA, and epithelial cell adhesion molecules (Chen et al., 2018; Katz et al., 2019). A different target is the deletion-mutation form of EGFR (known as EGFRvIII), expressed in a wide range of cancer tissues, including HCC tissues. It was identified as a suitable target by CAR T-cells in an *in vivo* model (female BALB/cA-nude mice) and an *in vitro* SMMC7721 cell line (expressing high levels of EGFRvIII). The researchers used CAR T-cells by applying the transposon system (piggyBac), and the results showed antitumor effects in both *in vivo* and *in vitro* models (Ma et al., 2020).

#### 5.2.7 Gastric Cancer

Gastric cancer (GC) is the fourth most commonly diagnosed type of cancer and the second cause of cancer-related death. Each year, the number of diagnosed patients is 990,000, of which 738,000 die (Machlowska et al., 2020). Different CAR T-cell targets against GC have been investigated, including folate receptor 1 (FOLR1) (Kim M. et al., 2018). HER2 is also a target in GC, and anti-HER2 CAR-T cells showed antitumor effects in MKN1 cells and mouse xenografts derived from a GC cell line with HER2 expression (Song et al., 2017). Several markers with diagnostic and functional importance have been studied as targets in GC, such as claudin 18.2 (CLDN 18.2), EpCAM, MUC1, CEA, EGFR2, natural-killer receptor group 2, member D (NKG2D), and MSLN. Other possible biomarkers that hold immense potential in GC include actin-related protein 2/3 (APR 2/3), desmocollin 2 (DSC2), B7H6 ligand, neuropilin-1 (NPR-1), cancer-related antigens CA-72-4 and CA-19-9, and anion exchanger 1 (AF1) (Zhang Q. et al., 2016). The use of anti-PSCA CAR T-cells on BGC-823, MKN-28, and KATO III GC cell lines and xenograft GC mouse models showed antitumor cytotoxicity post CAR T-cells peritoneal injection in mouse models resulted in tumor progression restriction (Wu et al., 2020).

#### 5.2.8 Colorectal Cancer

Colorectal cancer (CRC) incidence has reached 1.85 million cases worldwide. The mortality rate has reached more than 850,000 deaths per year, making it the third most common cause of death among cancer-related deaths (Biller and Schrag, 2021). The targeted antigens in CRC are NKG2D, CEA, EGFR, MUC1, HER2, and CD133 (Li et al., 2021). The membrane-bound guanylyl cyclase2C (GUCY2C) has been used as a CAR T-cell target. It showed antitumor activity in both human and syngeneic xenograft CRC mouse models and is expressed in the intestinal apical surface, epithelial cells, and a proportion of the hypothalamic neurons (Magee et al., 2016, 2018). Anti-EpCAM CAR T-cells used against CRC cells and models exhibited cytotoxic lysis of the targeted cells that secreted cytotoxic cytokines, including IFN-γ and tumor necrosis factor-alpha (TNF-α), resulting in tumor growth and development in xenograft mouse models (Zhang et al., 2019). The tumor-associated glycoprotein 72 (TAG-72) was used as a CAR T-cell target in CRC. It was infused in patients via the hepatic artery and intravenously. The CAR T-cells were confirmed in the blood, and trafficking to the tumor tissue was confirmed by tumor biopsy. The results showed antitumor effects of the anti-TAG-72 CAR T-cells. However, the metastatic deposits were resistant to these cells and escaped the immune attack (Hege et al., 2017). Doublecortin-like kinase 1 (DCLK1), involved in the epithelial-mesenchymal transition (TME) and tumor progression, is a novel target for CRC immunotherapy and anti-DCLK1 CAR T-cells resulted in cytotoxicity and secretion of IFN-γ after incubation with CRC cells in two. Higher secretion levels were observed in three-dimensional cultures (Sureban et al., 2019).

#### 5.2.9 Pancreatic Cancer

Pancreatic cancer (PaC) incidence has increased over the past few years, comprising 2% of all diagnosed malignancies and 5% of cancer-related deaths. Early diagnosis of PaC is challenging, and symptoms are not detectable at the early stages of the disease up to the advanced and metastatic settings. Most patients relapse, and the 5-year survival rate is 2% (Zhao and Liu, 2020). CXCR2-expressing CAR T-cells migrate more efficiently toward interleukin-8 (IL-8) and IL-8 containing TME, leading to a higher antitumor activity against αvβ6-expressing PaC xenografts (Whilding et al., 2019). B7-H3, also known as the CD276 antigen, was targeted by CAR T-cells in pancreatic adenocarcinoma *in vitro* and a metastatic xenograft mouse model, which proved efficacy (Du et al., 2019). Anti-CD133 CAR T-cells showed inhibitory activity against potential metastatic cells in HCC, colorectal carcinoma, and pancreatic carcinoma in phase I clinical trial (Wang et al., 2018). Other known antigens are being investigated for PaC CAR T-cell therapy, such as MUC-1 (Qu et al., 2004), fibroblast activation protein (FAP) (Tran et al., 2013), PSCA (Wu et al., 2020), CEA (Gansauge et al., 1996), mesothelin (Argani et al., 2001), CD24 (Jacob et al., 2004), and HER-2 (Komoto et al., 2009).

#### 5.2.10 Brain Cancer

The burden of the brain and central nervous system cancers is high. However, they occur rarely and comprise approximately 1.5% of all diagnosed cancers, 80% of all adult primary brain cancers are gliomas, and the relative 5-year survival rate is 22% in brain cancer (Sandler et al., 2021). Various targets of CAR T-cells in brain cancer have been studied, including EGFRvIII, which has several limitations, including adverse events such as dyspnea and hypoxia in patients. Another potential end is that the heterogenic expression of this target in glioma tumors might lead to the accumulation of resistant variants able to escape CAR T-cell therapy (Goff et al., 2019; Rutkowska et al., 2019). In a human pilot study where IL-13Rα2 was used as a target for CAR-T cells in treating glioblastoma via multiple intracranial infusions, the treatment was well-tolerated and antitumor activity was observed in patients (Brown et al., 2015). A study on HER2 as a target showed that the third generation HER2-specific CAR-T cells with enhanced activity combined with PD-1 blockade successfully eliminated glioblastoma cells (Shen et al., 2019). Additionally, HER2-specific CAR T-cells were infused in 17 patients. The infusion was well-tolerated, no dose-limiting toxicities were observed, and CAR T-cell persistence was detected for up to 12 months after infusion. No disease progression was observed during 24–29 months of follow-up (Ahmed et al., 2017). B7-H3 was targeted against glioblastoma in mouse models, and anti-B7-H3 Car T-cells led to significant tumor regression and extended survival (Tang et al., 2019). B7-H3 mRNA exists in all normal tissues, but the microRNAs inhibit its translation; however, conditions such as inflammation might elicit B7-H3 expression in these tissues, making them a target of anti-B7-H3 CAR T-Cells (Xu et al., 2009). The inducer of extracellular matrix metalloproteinase, known as CD147, is responsible for the degradation of the extracellular matrix, allowing for tumor growth, invasion, and metastasis (Xiong et al., 2014). CD147 expression in glioma is significantly higher than that in normal tissues, and its expression is correlated with patient prognosis (Yang et al., 2013; Li et al., 2017a). A phase 1 clinical trial was performed to evaluate the anti-CD147 effect in recurrent glioblastoma patients; however, low levels of this antigen in several normal tissues despite high levels in malignant tissues sparked concern (Riethdorf et al., 2006; Liao et al., 2011; Tseng et al., 2020). GD2 is also expressed in glioblastoma patient samples and cell lines (Golinelli et al., 2018). Anti-GD2 CAR T-cells exhibited cytotoxic activity against neuroblastoma cell lines *in vitro* and subcutaneously grafted cell lines in mouse models and successfully eliminated orthotopic patient-derived diffuse midline glioma xenograft models (Prapa et al., 2015; Mount et al., 2018). Chlorotoxin (CLTX) is found in the death stalker scorpion venom [(DeBin et al., 1993). CLTX was found to selectively bind to primary tumor cells, while it is hardly detectable in different types of normal brain tissues (Lyons et al., 2002). CLTX directed-CAR T-cells were generated to target glioblastoma, which exhibited antitumor activity in orthotopic xenograft mouse models (Wang D. et al., 2020). NKG2D receptors are expressed in glioblastoma stem-like cells (Flüh et al., 2018; Yang D. et al., 2019). Chemotherapy or radiotherapy upregulates the expression of the NKG2D ligand in glioblastoma cells; therefore, the combination of radiotherapy and anti-NKG2D CAR T-cells led to the prolonged survival of immunocompetent mice grafted with intracranial glioma cells (Weiss et al., 2018). In human differentiated glioblastoma cells and cancer initiation cells, and subcutaneous tumor models showed cellular eradication after CAR T-cell therapy; however, NKG2D-ligands on normal tissues are expressed under distress, which may result in human toxicity (Yang D. et al., 2019). In preclinical studies, various targets, such as carbonic anhydrase (CAIX), CD70, chondroitin sulfate proteoglycan 4 (CSPG4), erythropoietin-producing hepatocellular carcinoma A2 (EphA2), and trophoblast cell surface antigen 2 (TROP2) (Maggs et al., 2021).

#### 5.2.11 Malignant Pleural Mesothelioma

Malignant pleural mesothelioma (MPM) is an incurable, rare, and aggressive type of cancer that initiates at the serosal surfaces, including pleura, pericardium, peritoneum, and the vaginalis (in males), as a result of asbestos exposure, with an approximate survival of 8–14 months (Andujar et al., 2016; Carbone et al., 2019; Klampatsa and Albelda, 2020). In the United States, the incidence rate reached 3,200 diagnosed cases/year (Jane Henley et al., 2013), while in Europe, the cases are constant and are expected to have an increased trend between 2020 and 2025 (Carbone et al., 2019). MPM has three main histological mesothelioma subtypes: sarcomatoid, biphasic, and epithelioid (Yang et al., 2008). The disease is characterized by a significant therapeutic resistance and poor prognosis (Klampatsa and Albelda, 2020). Preclinical studies using mRNA electroporation exhibited potent anti-tumor effects (Zhao et al., 2010). In light of this, an initial study focusing (NCT01355695) on toxicity assessment was conducted using T-cells with transient expression of second-generation murine anti-mesothelin CAR containing CD3ζ and 41BB signaling domains (Maus et al., 2013; Beatty et al., 2014); in phase I safety trial none of the patients exhibited “on-target, off-tumor” toxicity post-infusion, and there was no evidence of clinical responses (Beatty et al., 2014; Klampatsa et al., 2017). However, an immediate anaphylactic reaction was observed in one of the patients post a delayed infusion of mesothelin CAR T cell, which was linked to the immunogenicity of the murine SS1 scFV used in the construction of CAR (Maus et al., 2013). After the safety confirmation of the transient CAR mesothelin expression, a second phase I clinical trial (NCT02159716) was conducted on 15 patients with mesothelioma, ovarian, and pancreatic cancer; the used CARs were expressing the same second-generation murine-based anti-mesothelin constructed using a lentiviral transduction vector (Haas et al., 2019). In this trial, two doses of T-cells were administered, and some cohorts used a lymphodepleting agent (cyclophosphamide). Although cyclophosphamide improved CART-meso expansion but did not enhance persistence beyond 28 days, the best overall response reported was stable disease in 11/15 patients (Haas et al., 2019). A third clinical trial (NCT03054298) was conducted using an active, fully human anti-mesothelin CAR and cyclophosphamide, administered via intravenous and intrapleural routes, respectively, to enhance the overall persistence and efficacy of CAR T-cells. In addition, researchers at Memorial Sloan Kettering Cancer Center are conducting a mesothelin-targeting CAR T cell trial to treat malignant pleural disease, including MPM (NCT02414269) based on preclinical studies of an orthotopic MPM mouse model. The study demonstrated that intrapleural administration of mesothelin CAR T-cell therapy was potent and had long-lasting antitumor efficacy (Adusumilli et al., 2014). The phase I/II clinical trial used CAR with human-derived anti-mesothelin scFV and CD3Z/CD28 signaling domain transduced by a retroviral vector; the CARs were administered via the intrapleural route in patients with primary and secondary pleural malignancies, with MPM patients being the main target population. A subset of the MPM patients had a subsequent administration of PD-1 checkpoint inhibitor (Pembrolizumab) to assess its efficacy in maintaining the prolonged activity of CAR T-cell therapy. Of the 27 patients who received cyclophosphamide, CAR T-cell therapy, and three doses of Pembrolizumab, 63% achieved either partial or complete response. Also, the CAR T-cells persisted and lasted for up to 42 weeks in the pleural fluid (Adusumilli et al., 2019).

### 5.3 HIV Infection

**FIGURE 4:**
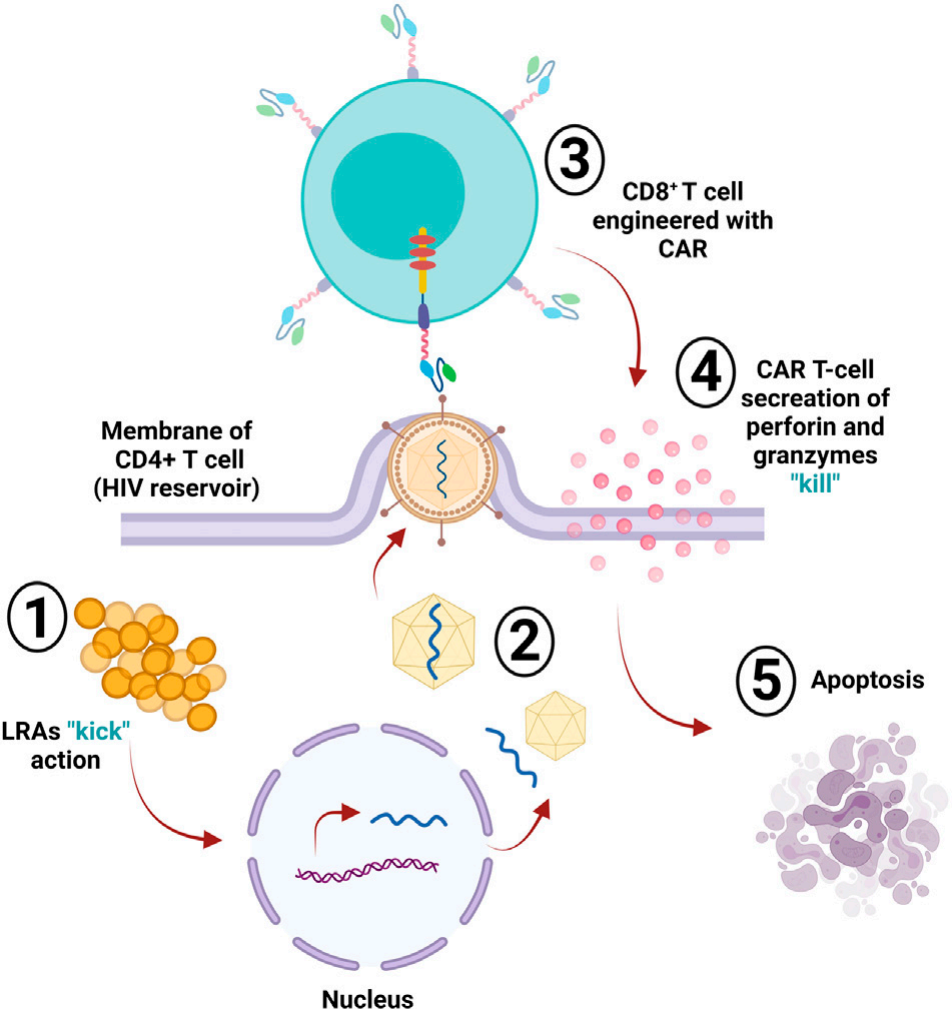
HIV reservoir eradication. The “kick and kill” strategy is used to eliminate latently infected cells (reservoir); the “kick” part of this strategy depends on latency reversal agents (LRAs), which induce the virus via transcriptional reactivation of the incorporated provirus within the infected cell. The infected CD4^+^ T-cell then starts producing and assembling the virus. Upon leaving the cell membrane, the engineered CD8^+^ CAR T-cell will detect the expressed viral antigens; then, the “kill” action occurs via the secretion of perforin and granzymes, sending the cell into apoptosis.

The human immunodeficiency virus (HIV) infects crucial cells in the human immune system, such as DCs, macrophages, and T helper cells (CD4^+^ T cells) (Cunningham et al., 2010). The deterioration of CD4^+^ T cells below critical levels renders the body susceptible to opportunistic infections (OIs) and the advancement of acquired immunodeficiency syndrome (AIDS) (Okoye and Picker, 2013). HIV-specific CD8^+^ cytotoxic T lymphocytes (CTLs) play an essential role in recognizing viral antigens presented by HLA class I and killing the infected cells, resulting in limited viral replication *in vivo*; however, CTLs fail to provide sustainable HIV replication control without the use of a combination antiretroviral therapy (cART) (Jones and Walker, 2016). The CTL responses still fail to clear the virus from the body, even when using cART to delay disease progression and increase life expectancy. HIV remains an incurable disease, and one of the main reasons behind the failure of the immune system to clear out HIV infection is the reduction or absence of HIV viral antigen expression on infected yet latent CD4^+^ T cells that act as viral reservoirs (Churchill et al., 2016). Viral reservoirs have been targeted by one strategy known as “kick and kill” or “shock and kill.” This approach suggests the induction of the virus from the latent cells to promote HIV eradication via cell death or by immune surveillance, which clears the viral reservoir (Kim Y. et al., 2018). However, this approach has been investigated in clinical trials using latency reversal agents (LRAs), and the results are not promising (Rasmussen et al., 2014; Spivak et al., 2014; Søgaard et al., 2015). CAR T-cells are a promising approach for targeting and killing HIV-expressing cells (Kuhlmann et al., 2018) for multiple reasons: 1) long-term immune surveillance provided by CAR T-cells: the effector function of peripheral-derived CAR T-cells has been reported to be maintained for 6 months (Kalos et al., 2011; Kochenderfer et al., 2012; Maude et al., 2014). Moreover, hematopoietic stem cell (HSPC)-derived CAR T-cells persist longer and provide constant production of CAR T-cells as observed in HIV/AIDS animal models (Zhen et al., 2017). Additionally, HSC-based CAR T-cells were found in several lymphoid tissues in the gut, bone marrow, and several lymph nodes, all of which represent the main replication sites in non-human primate (NHP), infected models, with simian-human immunodeficiency virus (SHIV) (Zhen et al., 2017). Moreover, the long-lived immunological memory provided by CAR T-cells can be reprogrammed and differentiated into central memory or effector T cells (Kawalekar et al., 2016). 2) The trafficking capability of CAR T-cells to various types of tissues, including the central nervous system, is considered a potential harbor for latent HIV (Marban et al., 2016). Penetration of the blood-brain barrier has been a difficult task for drugs; however, evidence of anti-CD19 CAR T-cell trafficking to brain tissues and cancer cell elimination supports the concept that CAR-T cells may effectively target HIV reservoirs in the brain tissues (Grupp et al., 2013; Maude et al., 2014). Homing receptors can be added to CAR T-cells to increase their presence in the B cell follicle, which is another important HIV reservoir difficult for CTLs to target (Haran et al., 2018). 3) The ability of CAR T-cells to target antigen in an MHC-independent manner helps in targeting HIV-infected cells and avoids viral downregulation of MHC-1 that leads to immune escape (Collins et al., 1998; Goulder and Walker, 1999; Wonderlich et al., 2011). The HIV CAR T-cell therapy targeted the primary HIV cellular receptor CD4, infused with CD3ζ signaling domain (CD4ζ) (Mitsuyasu et al., 2000; Walker et al., 2000; Deeks et al., 2002). The reason behind choosing CD4 as the reactive antigen in anti-HIV CAR T-cell design is its extensive targeting of all HIV isolates. Additionally, the binding sites of CD4 on the envelope protein are well preserved (Wang et al., 2019). The first generation CD4-based CAR-T cells have been tested in several clinical trials on HIV patients (Mitsuyasu et al., 2000; Walker et al., 2000; Deeks et al., 2002). The results showed a lack of durable control over viral replication; however, no treatment-associated toxicities were observed, and the persistence of modified cells continued for more than 10 years (Mitsuyasu et al., 2000). The first generation of CAR T-cells had certain impediments, such as limited *in vivo* expansion, susceptibility to apoptosis, and cytotoxicity (Heuser et al., 2003; Zhao et al., 2009). CAR T-cells were optimized into the second generation by adding costimulatory domains 4-1BB, resulting in 50-fold more compelling *in vitro* suppression of HIV replication than the previous generation (Leibman et al., 2017). *In vivo* studies showed that second generation CAR T-cells had superior expansion in response to the antigen, provided protection to CD4^+^ T-cells against HIV infection, and CD4 reduction was decreased compared to the CARs without costimulatory molecules (Leibman et al., 2017). The costimulatory domain 4-1BB is superior in reducing viral rebound than the CD28 domain after antiretroviral therapy (ART) and 4-1BB-induced T-cell perseverance in the absence of the antigen (Zhang et al., 2007; Leibman et al., 2017). Developing third generation CARs with multiple costimulatory molecules enhanced effector function, survival, and proliferation. It also enhanced tumor targeting and killing (Savoldo et al., 2011). Using the third generation CARs with CD3z-CD28-4-1BB as multiple domains, targeting the envelope glycoprotein GP120 (gp120) and anti-gp120 CAR T-cells in HIV infection showed increased effectiveness in lysing Env-expressing cells *in vitro* compared to CD4ζ CAR T-cells (Liu et al., 2016). Targeting HIV reservoirs by immune surveillance is difficult because of the ability of the virus to persist in various reservoirs and the lack of viral antigen expression in infected cells. The “kick and kill” strategy cause the transcription reactivation of the latently persistent provirus leading to viral antigen expression, making it detectable by the immune surveillance in ART-treated patients. The ‘kick” strategy can be achieved by potent latency reversal agents (LRAs). Clinical studies in animals showed that LRA was well tolerated *in vivo* and induced HIV expression (Marsden et al., 2017). Although LRAs induce the virus killing by the immune system, it is insufficient, and reservoir eradication is inefficient (Thorlund et al., 2017). The CAR T-cells can exhibit the “kill” response in this strategy along with LRAs; this combination is necessary for effective reservoir eradication (Bashiri et al., 2018) (Figure 4). The kill action in the human system shows that CTLs, either CD8^+^ or CD4^+^, induce apoptosis by cytolytic perforin and granzyme (Yasukawa et al., 2000).

**FIGURE 5:**
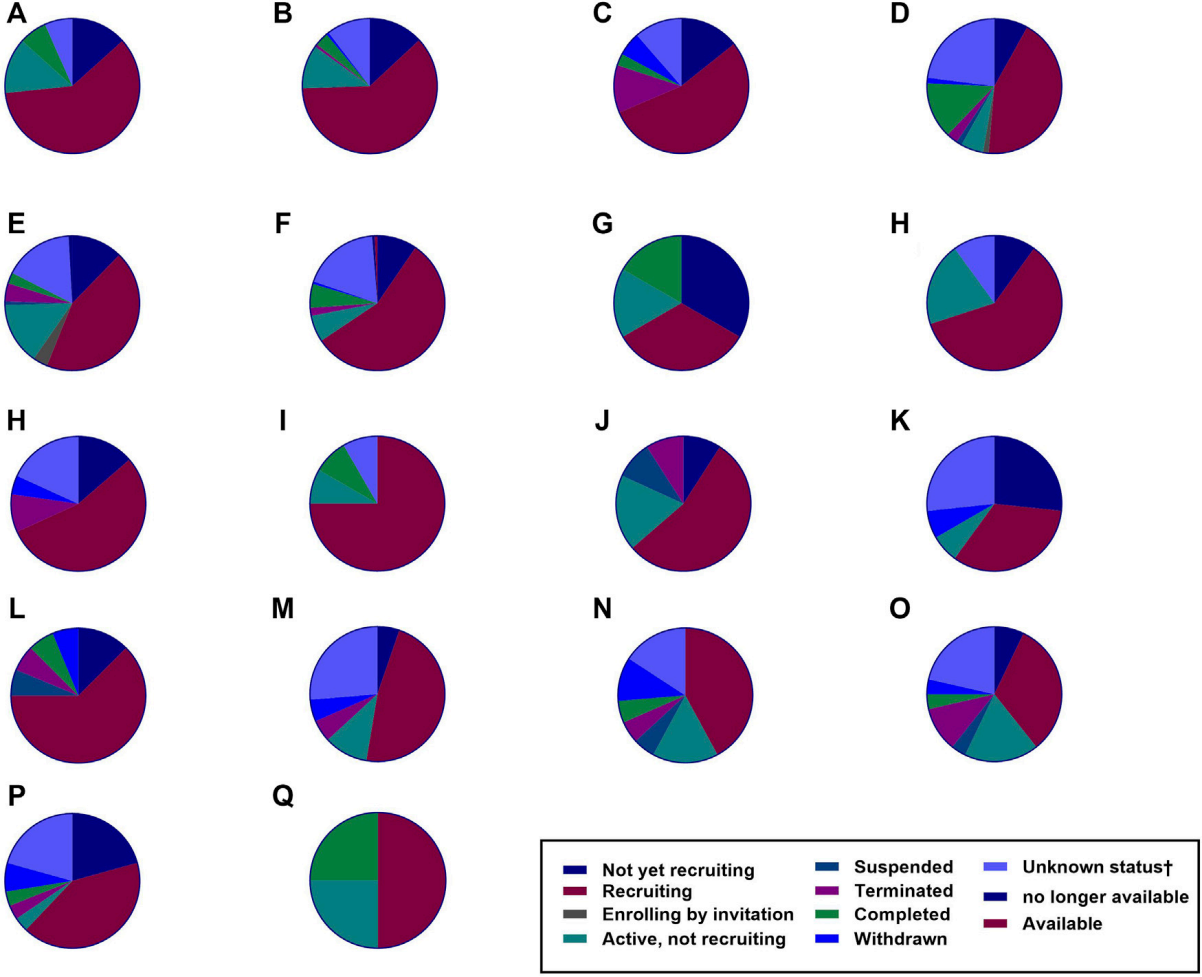
The number of clinical trials. Several clinical trials have been investigating various malignancies as recorded by ClinicalTrials.gov. Based on the data up to January 2022, the number of these clinical trials is rising. The figure shows the number of CAR T-cell therapy clinical trials for hematological malignancies, solid tumors, and HIV infection (total = 789). **(A)** Hodgkin’s lymphoma = 15 studies. **(B)** Acute myeloid leukemia = 35 studies. **(C)** Chronic lymphocytic leukemia = 74 studies. **(D)** Multiple myeloma = 114 studies. **(E)** Non-Hodgkin’s lymphoma = 153 studies. **(F)** Acute lymphoblastic leukemia = 157 studies. **(G)** Human Immunodeficiency Virus = 6 studies. **(H)** Prostate Cancer = 10 studies. **(I)** Brain Cancer = 12 studies. **(J)** Renal Cancer = 12 studies. **(K)** Colorectal Cancer = 15 studies. **(L)** Ovarian Cancer = 16 studies. **(M)** Lung Cancer = 22 studies. **(N)** Gastric Cancer = 19 studies. **(O)** Breast Cancer = 19 studies. **(P)** Pancreatic Cancer = 28 studies. **(Q)** Liver Cancer = 29 studies. **(R)** Malignant pleural mesothelioma = 4 studies.

CAR T-cell therapy has been considered a potential treatment against other infectious diseases such as those caused by opportunistic fungi, hepatitis B virus (HBV), hepatitis C virus (HCV), and cytomegalovirus (CMV), and the data gathered from pre-clinical trials have shown promising results (Seif et al., 2019). The number of clinical trials of CAR-T cell therapies is increasing, and their observations are constantly changing, as it is a very attractive field of research with remarkable potential (Figure 5). However, according to ClinicalTrials.gov, only 21 studies had results in January 2022 (Table 1).

**TABLE 1:** CAR T-cell clinical trials with recorded results from ClinicalTrials.gov.

Condition	Enrollment	Status	Antigen	Phase	Results	NCT
B- cell lymphoma	43	Active, not recruiting	Anti-CD19 CAR T-cells	Phase I/phase II	Complete remission (CR) of an assortment of the B-cell malignancies with durability for up to ≥3 years post 51% of anti-CD-19 CAR T-cell treatment with remission of 9 years and going. The adverse events were infrequent	(NCT00924326)
Metastatic melanoma and renal cancer	24	Terminated	Anti-VEGFR2- CAR T-cells	Phase I/phase II	Adverse events registered Grade 3 of 4 toxicity with a presentation of hypoxia, nausea, vomiting, hyperbilirubinemia, elevation in aspartate transaminase, and alanine transaminase. The study was terminated due to the absence of observed impartial responses	(NCT01218867)
Metastatic cervical, pancreatic, lung, ovarian, and mesothelioma cancers	15	Terminated	Anti-mesothelin CAR T-cell	Phase I/phase II	Adverse events were evident in this study, including anemia, constipation, thrombocytopenia, lymphocytopenia, and hypoxia. The study was terminated due to low and inadequate accrual	(NCT01583686)
Malignant gliomas	18	Completed	Anti-EGFRvIII CAR T-cells	Phase I/phase II	The pilot clinical trial failed and led to severe adverse events such as hypoxia, dyspnea, and multi-organ failure. In addition, the CAR T-cell intervention had no significant impact on the glioblastoma and resulted in its progression	(NCT01454596)
Refractory B-cell malignancies in children and young adults	53	Completed	Anti-CD19 CAR T-cells	Phase I	The feasibility and safety of this treatment were evident. The anti-leukemic activity was remarked in chemoresistance patients. High responses rate was observed post-infusion in patients. Central nervous system (CNS) trafficking and clearance were detected in two cases. Minimum cytokine release syndrome was CAR T-cells expansion correlated. Toxicities were reversible	(NCT01593696)
Relapsed or refractory CD19 positive chronic lymphocytic leukemia (CLL) and small lymphocytic lymphoma (SLL)	42	Completed	Anti-CD19 CAR T-cells	Phase II	Anti-leukemic activity and long persistence of tranced cells were seen in patients. Upon further investigation, findings suggest that patients who achieved complete response showed an increased mass of the Anti-CD19 CAR T-cells mitochondria, which contributed to cells expansion and persistence	(NCT01747486)
Adult B-cell Acute Lymphoblastic Leukemia (B-ALL)	82	Terminated	JCAR015 Anti-CD19 CAR T-cells	Phase II	The clinical trial failed to achieve significant results as five patients suffered from cerebral edema as an adverse event, resulting in death, and the study was terminated for safety reasons	(NCT02535364)
B-cell Malignancies (B-Cell Lymphoma, Non-Hodgkin’s Lymphoma)	27	Active, not recruiting	Anti-CD19 CAR T-cells. (Hu19-CD828Z)	Phase I	Patients had shown CR. This study suggested that enhancing the CAR T-cells design resulted in less neurotoxicity and CRS associated with low or mild cytokine production levels	(NCT02659943)
Multiple myeloma	6	Terminated	Anti-CD19 CAR T-cells. Post autologous stem cell transplantation (ASCT)	Phase II	No mortalities were reported. The serious adverse events were 1/6 patients suffered from CRS and upper respiratory tract infection (URI). The study was terminated due to administrative reasons	(NCT02794246)
B-cell Acute lymphoblastic leukemia in adults	1	Terminated	Anti-CD19 CAR T cells	Phase II	The patient died. The severe adverse events mentioned were paresthesia, encephalopathy, and gastric necrosis. The results were not discussed further, and the study was terminated due to admirative reasons	(NCT02935543)
Glioblastoma and gliosarcoma	3	Terminated	Anti- EGFRvIII CAR T-cells	Phase I	The mortalities were 3/3. The adverse events were confusion and generalized muscle weakness in 1/3. The study was terminated because the funding was not sufficient	(NCT02664363)
Multiple myeloma	12	Terminated	AUTO2 (APRIL CAR T-cells)	Phase I/phase II	The study mortalities were 6. Some patients have severe adverse events, including Acute myocardial infarction (AMI), pyrexia, lung infection, decreased neutrophil count, hypocalcaemia, metaplastic breast carcinoma, headache, and dyspnea. The study was terminated as the preliminary efficacy post-treatment was insufficient to guarantee further development	(NCT03287804)
B Cell Acute Lymphoblastic Leukemia (ALL)	23	Completed	AUTO3 (CD19/22 CAR T-cells)	Phase I/phase II	The mortality rate was 61.6% among patients who received high infusion doses; serious adverse events were anemia, febrile neutropenia, thrombocytopenia, pyrexia, cellulitis, encephalopathy, and seizure	(NCT03289455)
Relapsed/refractory B-cell malignancies	26	Active, not recruiting	Anti-CD20/19-CAR T-cells	Phase I	The results of this study suggest that the favorable infusion dosage is 2.5 × 10^6^ cells/kg providing low toxicity and high efficacy in city profile and sustained efficacy at a dose of 2.5×10^6^ cells per kg for relapsed, refractory B cell non-Hodgkin’s lymphoma (NHL) and chronic lymphocytic leukemia (CLL) patients	(NCT03019055)
Relapsed/Refractory Multiple Myeloma	17	Active, not recruiting	KITE-585 CAR T-cells	Phase I	The overall mortality rate was 62.5%, and the adverse events were chest pain and hypoxia	(NCT03318861),
Advanced Lung Cancer	1	Terminated	Anti-PD-L1 CAR T-cells	Phase I	The patient developed severe CRS, which caused interstitial pneumonia disease. The study was terminated due to serious adverse events	(NCT03330834),
Acute Myeloid Leukemia (AML) Multiple Myeloma (MM)	8	Terminated	Anti-CD44v6 CAR T-cells	Phase I/phase II	The patients had adverse events of pyrexia, anemia, neutropenia. The study was terminated due to low patient recruitment and a lower-than-expected proportion of myeloma and leukemia expressing CD44v6. The study failed to be completed in a clinically relevant time frame	(NCT04097301)
CD19^+^ Diffuse Large B-cell Lymphomas Follicular Lymphomas Mantle Cell Lymphomas	12	Completed	Anti-CD19 CAR T-cells	Phase I/phase II	Serious adverse events included optic disorder, fever, hyperbilirubinemia, CRS, sepsis, hypercalcemia, delirium, acidosis, hypoxia, pleural effusion, non-cardiac related chest pain, and rash	(NCT02650999)
DLBCL Neurotoxicity Syndromes	25	Terminated	Evaluation of the Safety and Efficacy of Defibrotide in the Prevention of Chimeric Antigen Receptor-T-cell-associated Neurotoxicity	Phase II	Patients had febrile neutropenia, atrial fibrillation, myocardial infarction, asthenia, pyrexia, CRS, decreased appetite, neurotoxicity, tumor lysis syndrome, transient ischaemic attack, confusion state, pleural effusion, pulmonary embolism, and hypotension. The study was terminated because unplanned interim assessment on the first 20 efficacy evaluable patients was unlikely to meet the primary endpoint	(NCT03954106)
Relapsed or Refractory Neuroblastoma	17	Completed	Anti-GD2 CAR T-cells, (1RG – CART)	Phase I	Hypotension, capillary leak syndrome, neurological symptom, headache, hyponatremia, pyrexia, tachycardia, febrile neutropenia, and coagulopathy. Only 12 patients were subjected to therapy as two were withdrawn due to progressive disease, one died, and one withdrew the consent for the trial	(NCT02761915)
Myeloma-Multiple Myeloma, Plasma-Cell	13	Completed	Anti-SLAMF7 CAR T-cell	Phase I	Serious adverse events included CRS sinus tachycardia and fever	(NCT03958656)

It is worth of mentioning that CAR T-cells potentials were recently applied against cardiac fibrosis (heart tissue stiffening and scarring). Rurik et al. were capable of designing an immunotherapy strategy to generate transient CAR T-cells able to identify fibrotic cells in the heart through injecting CD5-targeted lipid nanoparticles encompassing the needed mRNA to reprogram T lymphocytes, therapeutic CAR T-cells were successfully generated inside the body (*In vivo*). The heart disease in mouse model was analyzed and revealed that this approach has indeed succeeded in fibrosis reduction and cardiac function restoration (Rurik et al., 2022).

## 6 FDA Approved CAR T-Cells Therapies

### 6.1 Axicabtagene Ciloleucel (YESCARTA™)

The first Food and Drug Administration (FDA) approved CAR T-cell therapy, axicabtagene ciloleucel (YESCARTA™) from Kite Pharma approved in 2017, comprises autologous genetically modified T cells designed to produce CAR protein targeting CD19 expressing normal and malignant cells (Papadouli et al., 2020). It is used to treat adult large B-cell Lymphoma after two or more lines of systemic therapy, including DLBCL, high-grade B-cell lymphoma, primary mediastinal large B-cell lymphoma, and DLBCL arising from follicular lymphoma. The approval of this drug was based on a single-arm multicenter clinical trial (ZUMA-1; NCT02348216) conducted on 108 patients diagnosed with aggressive B-cell non-Hodgkin’s lymphoma. The selection criteria were occurrence of refractory disease post a recent therapy or relapse post autologous hematopoietic stem cell transplantation within a year. The patients underwent lymphodepletion before receiving a single infusion of axicabtagene ciloleucel. The efficacy was evaluated in 101 patients as follows: ORR 72%, with a complete remission rate (CR) of 51%, the duration of response (DOR) was longer in patients with CR than in patients with partial remission (PR). The median DOR was not reached after 7.9-months (median follow-up). The estimated DOR was 2.1 months. Most common grade 3 (with incident ≥10%) adverse events occurred including fever, febrile neutropenia, encephalopathy, CRS, hypoxia, and hypotension; 25% exhibited severe adverse events, including neurotoxicity, CRS, serious infections, and prolonged cytopenia. In some patients, CRS and neurotoxicity were fatal. The FDA approved axicabtagene ciloleucel with recommendations of mitigation strategy and risk evaluation. The recommended dosage was 2 × 10^6^ viable CAR-positive T cells/kg of body weight, following lymphodepletion chemotherapy by (Flu/Cy) (Neelapu et al., 2017a). In March 2021 (Yescarta, axi-cel), the FDA approved another directed CD19 T-cell therapy to treat adult r/r follicular lymphoma after two lines of therapy. The approval was based on collected data from a single-arm, open-label phase II clinical trial (ZUMA-5; NCT03105336). The clinical trial had 81 participants. The results were: ORR 91%, with CM of 60%, the median DOR was not reached within a year of CM rate of 76.2%, patients who underwent leukapheresis (*n* = 123) experienced a ORR of 89% with CM rate 62%. CRS (grade ≥ 3, 10%) occurred in 88%, and neurotoxicity occurred in 51% of all patients with non-Hodgkin’s lymphoma (Colombo et al., 2021).

### 6.2 Tisagenlecleucel (KYMRIAH™)

The second FDA approved CAR T-cell therapy, tisagenlecleucel (KYMRIAH™) from Novartis pharmaceuticals approved in 2018, is a genetically modified autologous T-cell immunotherapy (CD19 directed) for adult patients with r/r large B-cell lymphoma post two or more lines of systemic therapy, including high-grade B-cell lymphoma DLBCL, and DLBCL arising from follicular lymphoma. The approval was based on phase II of a single-arm, open-label, multicenter clinical trial (JULIET; NCT02445248) conducted on adults with r/r DLBCL and DLBCL arising from follicular lymphoma (Schuster et al., 2019). The criteria included a condition that the subject must at least undergo two prior therapy lines with rituximab and anthracycline or have relapsed after autologous hematopoietic stem cell transplant. Patients had a single tisagenlecleucel infusion after the completion of lymphodepleting chemotherapy. The clinical trial had 68 eligible patients out of 115, and the outcomes were 50% ORR with a 32% CR rate. With a median follow-up time of 9.4 months, patients with the best overall response CR had longer DOR than that of patients with PR. Patients with CR estimated median DOR of (10.0 months) was not reached, while the estimated median DOR among PR patients was 3.4 months. The most common adverse events in 20% of the patients included CRS, pyrexia, nausea, infections-pathogens unspecified, fatigue, diarrhea, headache, edema, and hypotension. The recommended dose of tisagenlecleucel for adults with r/r DLBCL was 0.6–6.0 × 10^8^ CAR-positive viable T-cells (Schuster et al., 2019).

### 6.3 Brexucabtagene Autoleucel (TECARTUS™)

Accelerated approval of brexucabtagene autoleucel (TECARTUS™) was granted by FDA in July 2020; this immunotherapy comprises autologous genetically modified T cells (CD19-directed) for the treatment of adult patients with r/r mantel cell lymphoma (MCL) (Wang M. et al., 2020). The clinical trial behind the approval was a multicenter, single-arm, and open-label (ZUMA-2; NCT02601313) trial. Seventy-four patients diagnosed with MCL were subjected to this study. These patients previously received anthracycline or bendamustine-containing chemotherapy, anti-CD20 antibody, and Bruton tyrosine kinase inhibitor. After completing lymphodepleting chemotherapy, patients received a single infusion of brexucabtagene autoleucel. Sixty out of 74 patients evaluated for efficacy in a minimum duration of 6 months follow-up showed 87% ORR, with a CR rate of 62%. The estimated DOR was not reached (0–29.2 months) after a median DOR of follow-up (8.6 months). Among all 74 patients who underwent leukapheresis, the ORR was 80%, and CR was 55%. The most common adverse reactions with grade 3 or higher (≥10%) included hypoxia, encephalopathy, leukopenia, anemia, neutropenia, thrombocytopenia, hypotension, hypophosphatemia, hypertension, hyponatremia, pyrexia, infection-pathogen unspecified, lymphopenia, hypocalcemia, and pneumonia. Due to the fatal or life-threatening neurotoxicity and CRS, the FDA approval came with risk evaluation and mitigation strategies. The recommended dose of brexucabtagene autoleucel was a single IV infusion of (2 × 10^6^ – 2 × 10^8^)CAR-positive viable T-cells/kg body weight post lymphodepleting chemotherapy of (Flu/Cy) (Wang M. et al., 2020).

In 2021 brexucabtagene autoleucel was approved to treat adult patients with r/r B-cell precursor ALL based on the data gathered from a phase II clinical trial (ZUMA-3; NCT02614066) (Shah et al., 2021). The study had 125 participants diagnosed with r/r B-cell precursor ALL. Patients received a single infusion of brexucabtagene autoleucel post completion of lymphodepleting chemotherapy. The outcomes included CR within 3 months post-infusion. Fifty-four patients were evaluable for efficacy, 28 achieved CR within 3 months with a median follow-up of 7.1 months, the CR median duration was not reached, and the CR duration for more than half of the patients was estimated to exceed 12 months. In 92% of patients, CRS occurred (≥grade 3, 26%); neurotoxicity occurred in 87% (≥Grade 3, 35%); most common adverse events were hypotension, CRS, encephalopathy, fever, chills, headache, rash, edema, nausea, tachycardia, febrile neutropenia, musculoskeletal pain, hypoxia, diarrhea, tremor, constipation, infection with an unspecified pathogen, vomiting and decreased appetite. The recommended dose was a single IV infusion of brexucabtagene autoleucel (1 × 10^6^–1 × 10^8^) of CAR-positive viable T-cells/kg body weight preceded by (Flu/Cy) lymphodepleting chemotherapy (Shah et al., 2021).

### 6.4 Lisocabtagene Maraleucel (BREYANZI™)

In February 2021, lisocabtagene maraleucel (BREYANZI™) from Juno Therapeutics was approved by FDA for the treatment of adult patients with r/r large B-cell lymphoma after two or more lines of systemic therapy, including high-grade B-cell lymphoma, DLBCL, primary mediastinal large B-cell lymphoma, DLBCL arising from indolent lymphoma, and follicular lymphoma grade 3B (Abramson et al., 2020). Lisocabtagene maraleucel is a CD19- directed CAR T-cell immunotherapy comprised of autologous genetically modified T cells that produce CAR protein able to identify and eradicate CD19-expressing normal and malignant cells. The immunotherapy efficiency was evaluated in a single-arm, open-label, multicenter trial (TRANSCEND, NCT02631044); 192 patients underwent lymphodepleting chemotherapy before infusion. The outcomes included 73% ORR, with a CR rate of 54%, and the median time of first response was 1 month; 104/192 patients had CR, which lasted at least 6 months (65%), and some patients (62%) had a remission that lasted at least 9 months. The DOR was 16.7 months in patients who achieved CR; and the patients with PR had 1.4 DOR. Adverse events included CRS in 46% of the patients (grade 3 or higher, 4%); neurotoxicity occurred in 35% (grade 3 or higher, 12%). Three patients encountered fatal neurotoxicity. Other grade 3 or higher adverse events were prolonged cytopenia (31%) and infections (19%). Due to the fatal and life-threatening neurotoxicity and CRS, the FDA approval came with recommendations of risk evaluation and mitigation strategies. The recommended regimen was a single dose of 50–110 × 10^6^ CAR-positive viable T-cells with a ratio of 1:1 of CD4 and CD8 components, intravenous (IV) infusion following (Flu/Cy) lymphodepletion (Abramson et al., 2020).

### 6.5 Idecabtagene Vicleucel (ABECMA™)

On March 2021, idecabtagene vicleucel (ABECMA™) from Bristol Myers Squibb was approved as the first cell-based immunotherapy for adult patients with r/r multiple myeloma after four or more preceded lines of therapy, including an anti-CD38 monoclonal antibody, an immunomodulator, and a proteasome inhibitor (Munshi et al., 2021). It is an autologous genetically modified B-cell maturation antigen (BCMA)-directed CAR T-cell therapy. In a multicenter study (NCT03361748), a total of 127 patients with r/r multiple myeloma were included to evaluate the safety and efficacy of the idecabtagene vicleucel; all patients received three (88% had received four or more) lines of antimyeloma therapies. In addition, 100 had received idecabtagene vicleucel with a dosage range of 300–460 × 10^6^ of CAR-positive T-cells. The results showed a 72% ORR and a CR rate of 28%. Approximately 65% of patients had CR for at least 12 months. The most common adverse events included CRS, neurotoxicity, macrophage activation syndrome, prolonged cytopenia. Moreover, infection, fatigue, hypogammaglobulinemia, and musculoskeletal pain were designated as common side effects. Idecabtagene vicleucel was approved with recommendations of risk evaluation and mitigation strategies. The healthcare facility that houses this therapy must be specially certified to recognize and manage neurotoxicity and CRS. FDA called for a post-marketing observational study conducted by the manufacturer involving the patients treated with idecabtagene vicleucel (Munshi et al., 2021).

### 6.6 Ciltacabtagene Autoleucel (CARVYKTI ™)

The most recently FDA approved CAR T-cell therapy, in February 2022, is ciltacabtagene autoleucel (CARVYKTI™) from Janssen Biotech, Inc. This drug was approved for the treatment of r/r multiple myeloma post four or more prior lines of therapy including an anti-CD38 monoclonal antibody, an immunomodulatory agent (IMiD), and a proteosome inhibitor (PI). It is a genetically modified autologous CAR T-cell therapy directed by B-cell maturation antigen (BCMA). In a multicenter study CARTITUDE-1 (NCT03548207) ciltacabtagene autoleucel safety and efficacy of were evaluated in 97 patients with r/r multiple myeloma who presented disease progression post their last chemotherapy regimen; 82% of the patients had received four or more prior lines of antimyeloma therapy. The dosage of ciltacabtagene autoleucel given to patients was falling within the range of 0.5–1.0 × 10^6^ viable CAR-positive T-cells/kg body weight. According to the International Myeloma Working Group Uniform Response Criteria for Multiple Myeloma, the efficacy was evaluated by an Independent Review committee based on the overall ORR and DOR response. The ORR 97.9%, and a median DOR of 21.8 and 12 months median duration of follow up. Most commonly observed adverse reactions of ciltacabtagene autoleucel were CRS, fatigue, hypogammaglobulinemia, pyrexia, musculoskeletal pain, nausea, infection, diarrhea, coagulopathy, encephalopathy, headache, vomiting, and constipation. Moreover, recommended dosage of (CARVYKTI™) ranges from 0.5–1.0 × 10^6^ to a maximum dose of 1 × 10^8^ viable CAR-positive T-cells/kg of body weight per single infusion. The approval of (CARVYKTI™) is restricted by a risk evaluation and mitigation strategy necessitating healthcare facilities that houses this therapy and their associated clinicians to be specially certified to recognize and manage neurotoxicity and CRS. FDA called for a post-marketing observational study conducted by the manufacturer involving the patients treated with ciltacabtagene autoleucel (Berdeja et al., 2021).

## 7 Limitations and Solutions for CAR T-Cells

The CAR T-cell technology has immense potential. Current clinically approved CAR T-cell therapies are KYMRIAH™ for ALL and DLBCL; YESCARTA™ for DLBCL and follicular lymphoma; TECARTUS™ for mantle cell lymphoma; BREYANZI^®^ for DLBCL and follicular lymphoma; and ABECMA^®^ for MM. Unfortunately, all these approved CAR-T cell products exert serious but clinically manageable adverse effects such as cytokine release syndrome and neurotoxicity (Zhao Z. et al., 2018; Zheng et al., 2018). Notably, the delay in approving CAR T-cell therapies targeting other diseases has the following structural limitations.

### 7.1 Tumor Antigen Escape

Single antigen-targeting CAR-T cells might face tumor resistance after the initial high response rate. The decline in response and increase in resistance is due to partial or complete loss of target antigen expression. Tumor cells escape killing by encouraging mutations in the antigen-coding gene, leading to the downregulation of expression of alternative antigens that lack the antigen epitopes targeted by CAR T-cells (Majzner and Mackall, 2018; Sterner and Sterner, 2021). One strategy to overcome this hurdle is to design T cells equipped with two or more CARs to target multiple TAAs, suggesting that the escape mechanism would require mutation of several genes instead of one by engineering CARs with multi-specific targets such as bicistronic CAR T-cells, tandem CAR T-cells, co-administered CAR T-cells, or co-transduction CAR T-cells. However, finding more than one TAA in one tumor targeted by CAR T-cells may prove challenging in some malignancies, with respect to safety and effectiveness (Hegde et al., 2013; Jackson and Brentjens, 2015). In addition, the use of lymphodepleting agents before the adoptive T-cell transfer can enhance epitope spreading, leading to more specific antigen recognition (Cui et al., 2009). Additionally, combining CAR T-cell therapy with checkpoint inhibitors (Gargett et al., 2016; Li et al., 2017b; Heczey et al., 2017; Adusumilli et al., 2021), radiation (Weiss et al., 2018), vaccines (Slaney et al., 2017; Tanaka et al., 2017), other immune agonists (Khalil et al., 2016; Majzner et al., 2017) might also contribute to epitope spreading and immune escape restriction (Majzner and Mackall, 2018).

### 7.2 On-Target Off-Tumor

One of the most observed toxicities in CAR T-cell therapy is the “on-target-off-tumor,” where the normal tissues express the same targeted antigen on the malignant tissues at variable levels, leading to a direct attack from CAR T-cells against the normal tissues and eventually resulting in toxic effects that can be detrimental (Sun et al., 2018). To overcome this roadblock, using affinity-tune CARs to recognize tumor cells that have increased density of surface antigens and preventing the involvement with normal tissues that express low-density surface antigens has been suggested (Zhao et al., 2009). This strategy can be executed by altering the binding region of scFV via mutagenesis or via the recombination of both heavy and light chains (Carter et al., 1992; Drent et al., 2017). Another potential avenue for solid tumors is to target tumor-restricted post-translational modifications, such as overexpression of truncated O-glycans such as Tn (GalNAca1-O-Ser/Thr) and sialyl-Tn (STn) (NeuAca2–6-GalNAca1-O-Ser/Thr) (Steentoft et al., 2018). Another suggested approach is CAR T-cell local administration to the disease site, which might contribute to the limitation of “on-target-off-tumor” toxicity as the on-target activity is focused on the malignant tissue, and the normal tissue interaction is disregarded (Sterner and Sterner, 2021). Inducible CAR-T cell products based on engineered synthetic Notch receptors are also being explored to mitigate the on-target off-tumor associated toxicities (Roybal et al., 2016).

### 7.3 Trafficking and Tumor Infiltration

One of the significant inadequacies in using CAR T-cell therapy in solid tumors is the ability of these cells to traffic and infiltrate the tumor because both immunosuppressive TME and physical barriers of tumor such as stroma restrain mobility and diffusion of CAR T-cells. The proposed approach uses the local administration as the delivery route, which disregards the need for the cells to traffic to the disease site (Sterner and Sterner, 2021). Another strategy developed to overcome the trafficking issue is the addition of chemokine receptor expression on CAR T-cells that match and respond to chemokines expressed by targeted tumors (Whilding et al., 2019). The physical barrier of the stroma mainly comprises an extracellular matrix with a primary component of heparin sulfate proteoglycan (HSPG). Upon its degradation, CAR T-cells can reach the tumor (Zhang B.-L. et al., 2016). Engineered CAR T-cells with heparinase expression have been shown to degrade HSPG, leading to enhanced tumor infiltration and elimination (Caruana et al., 2015). Likewise, fibroblast activation protein (FAP) was also targeted by CAR T-cells in animal models, which increased cytotoxic function by reducing the number of tumor fibroblasts (Wang et al., 2014).

### 7.4 Immunosuppressive Microenvironment

In the TME, several tumor-infiltrating cells contribute to immunosuppression, including MDSCs, regulatory T cells (Tregs), and tumor-associated macrophages (TAMs) (Quail and Joyce, 2013). These infiltrates and tumor cells contribute to the production of tumor-supporting growth factors, chemokines, and cytokines, and the antitumor immunity declines because of immune checkpoint proteins such as CTLA-4 or PD-1. Weak CAR T-cell responses could be regarded as a poor T-cell expansion and limited persistence period, indicating that the development of T-cell exhaustion is prompted by co-inhibitory pathways (Yin et al., 2018). Consequently, the combination of CAR-T cells with immunotherapy and checkpoint blockade is thought to be the next cutting-edge immunotherapy approach because it provides two major elements to secure strong immune responses: CAR T-cells provide tumor penetration and PD-1/PDL1 blockade to guarantee sustained and persistent T-cell function (June et al., 2018; Grosser et al., 2019). Recently, CAR-T cells have been engineered to be robustly resistant to TME immunosuppressive factors such as TFG-β-mediated inhibitory signals (Kloss et al., 2018). Furthermore, CAR T-cell engineering includes the addition of immunostimulatory signals such as stimulatory cytokines capable of increasing survival, proliferation, and antitumor activity while re-equalizing TME (Chmielewski et al., 2014). Various studies have been investigating numerous cytokines to create “armored CARs.” The studies that focused on proinflammatory cytokines apart from concentrating on inhibitory signals have depended on IL-12 secretion (Koneru et al., 2015), expression of IL-15 (Krenciute et al., 2017), and the redirection of immunosuppressive cytokine signaling (e.g., IL-4) towards proinflammatory cytokines (Mohammed et al., 2017).

### 7.5 CAR T-Cell-Associated Toxicities

T-cell therapy has been one of the most groundbreaking tools in cancer treatment; however, toxicities and associated fatalities have limited this approach’s applications. To date, the characterization of the toxicities associated with CAR T-cell therapy has been broadly studied in patients receiving FDA-approved CAR T-cell therapy such as anti-CD19 CARs (Sterner and Sterner, 2021). Several factors determine the occurrence and intensity of (CRS), hemophagocytic lymphohistiocytosis (HLH), macrophage activation syndrome-like activation (MAS-L) (HLH/MASL), and immune effector cell-associated neurotoxicity syndrome (ICANS), including tumor type, specific target, and CAR design (Roex et al., 2020).

The most frequent acute toxicity associated with CAR T-cell therapy is the CRS; the cytokines involved are produced either by the infused CAR T-cells or by the CAR T-cell-responding immune cells such as macrophages. These cytokines include TNF-α, several interleukins such as IL-6, IL-2, -IL-2α, IL-8, IL-10, and IFN-γ, which were elevated in the patient’s serum. Also, patients with severe CRS experience high-grade pyrexia, which can develop into an uncontrolled systemic inflammatory response with circulatory shock requiring vasopressors, vascular leakage, disseminated vascular clots, tachycardia, hypotension, hypoxia, and multi-organ system dysfunction. The severity of the CRS was correlated with the type of cytokines detected in the serum (Brudno and Kochenderfer, 2016; Shimabukuro-Vornhagen et al., 2018). Organ dysfunction can be reversed in most patients once CRS signs are recognized and managed early (Morris et al., 2021). Management of CRS using supportive care includes antipyretics, blood components transfusion, intravenous fluids, vasopressors, monoclonal antibodies (tocilizumab) used against the IL-6 receptor, and steroids in high-grade CRS. Both tocilizumab and steroids can control CRS in most cases. However, resistant CRS can also develop where the symptoms persist regardless of supportive treatments in a minority of patients, putting them at a high mortality risk (Yang X. et al., 2019).

ICANS is another common toxicity occurring after CAR T-cell infusion and is associated with treatment-related morbidity. However, the exact mechanism underlying the manifestation of neurologic toxicity remains indistinct. CAR T-cell facilitated inflammation-causing endothelial activation and disruption of the blood-brain barrier may play a central role (Holtzman et al., 2021). ICANS manifestation begins with toxic encephalopathy, aphasia, dysphasia, impaired motor function, and drowsiness. In severe cases, more severe symptoms occur, such as seizures, motor weakness, cerebral edema, and coma, most patients experiencing ICANS had earlier CRS that had subsided. Therefore, CRS could be considered an early sign of ICANS. Concurrence between ICANS and CRS occurs less frequently. ICANS is also reversible in patients who do not develop permanent neurological deficits (Morris et al., 2021). Management of ICANS aims to reduce the inflammatory response, which could be achieved by using Siltuximab (IL-6 antagonist), which prevents continuous IL-6 translocation across the blood-brain barrier (Gust et al., 2017). A high dose of corticosteroids shows sound central nervous system (CNS) penetration (Neelapu et al., 2017b). The use of levetiracetam or other antiepileptic agents can also be considered an option for treating severe neurological dysfunction as prophylaxis for seizures (Pehlivan et al., 2018). Additional studies are required to understand the mechanism underlying ICANS manifestation, associated risk factors, and optimal management required for CAR-T cell infusion.

HLH is a rare condition characterized by fever, hyperferritinema, splenomegaly, hypertriglyceridemia, coagulopathy, and cytopenia due to improper immune activation and cytokine release (Risma and Jordan, 2012a). In patients with low-grade CRS, HLH can occur; however, severe CRS might evolve into HLH. Thus, clinicians must pay attention to this condition to prevent fatal outcomes HLH/MAS post CAR T-cell therapy in association with CAR T-cell induced toxicities (CARTOX) score, which includes serum ferritin levels >10.000 ng/ml and one of the following: oliguria grade ≥3 or elevated serum creatinine grade ≥3, pulmonary edema, elevation in serum bilirubin, aspartate aminotransferase or alanine aminotransferase grade ≥3, and incidence of hemophagocytosis bone marrow (Mei et al., 2018). Management of HLH/MAS as mentioned in CRS and ICANS with anti-IL-6 agents and corticosteroids can be used. However, if the condition persists for almost 48 h, other interventions, such as intrathecal cytarabine and etoposide, especially in neurotoxicity-associated HLH (Neelapu et al., 2017b).

Several recommendations have been proposed to attenuate the toxicities resulting from CAR T-cells: 1) to ensure that the therapeutic efficacy is valid and no toxic overshooting of cytokines is occurring by monitoring the CAR T-cell activation threshold post-infusion. Activation of CAR T-cells is influenced by several factors, including tumor antigen expression levels on malignant cells, the affinity of the antigen-binding domain to target epitope, tumor burden, costimulatory elements of CARs (van der Stegen et al., 2015; Milone and Bhoj, 2018); 2) to achieve low affinity of the antigen-binding domain to ensure selectivity for tumors with high expression levels of targeted antigen; 3) hinge-region and transmembrane region modifications and optimization to control cytokine secretion levels and keep them within the therapeutic window as seen in anti-CD19 CAR T-cells where no CRS or ICANS were observed (Ying et al., 2019); 4) costimulatory domain can be customized based on tumor burden, tumor antigen binding domain engagement, antigen density, and toxicity concerns. Evidence suggests that 4-1BB costimulatory domains show lower toxicity risk, lower T-cell expansion levels, higher T-cell endurance. In contrast, CD28 costimulatory domains are associated with CAR T-cell onset rapid activation and consequent exhaustion. These properties make 4-1BB domains more preferable in cases of high disease burden or/and high tumor antigen density, and in cases of low surface antigen density or/and low-affinity antigen-binding domain CARs with CD28 costimulatory domains are more preferable (Salter et al., 2018); 5) CARs immunogenicity can be decreased by modifying hinge region and/or transmembrane domain, which also contributes to CAR T-cell persistence improvement (Jonnalagadda et al., 2015; Sommermeyer et al., 2017); 6) neutralization of GM-CSF to overcome CRS and neurotoxicity, tyrosine hydroxylase inhibition by metyrosine or deletion of this enzyme in a myeloid cell-specific manner resulted in catecholamine and cytokine levels reduction (Staedtke et al., 2018), use of IL-1 antagonists to reduce neuroinflammation (Giavridis et al., 2018); 7) use of “off-switch” or suicide gene strategies to encourage selective elimination of CAR T-cells at the commencement of adverse events under a secondary agent control. However, the slow onset of antibody-mediated depletion limits the efficacy of this approach, especially in patients who require immediate reversal during acute and severe cytokine toxicities; therefore, faster switches such as inducible cas9 were developed and proved to deplete 90% of CAR T-cells within 30 min (Di Stasi et al., 2011; Jones et al., 2014). Engineering CAR T-cells with CD20 full-length expression or CD 20 mimotopes, which deplete CAR T-cells post rituximab treatment (Philip et al., 2014), use of switch off CARs (SWIFF-CARs) (Juillerat et al., 2019). The most significant limitation in utilizing the suicide gene strategy is the sudden cessation of therapy in rapidly progressing diseases, making this strategy a last resort. However, recently, the use of TKIs, which inhibit proximal TCR signaling kinases and suppress T cell activation (dasatinib), provide temporary inhibition of CAR T-cells. CAR T-cell activity would resume after toxicity has subsided (Sterner and Sterner, 2021). Additional studies are required to overcome all toxicities without affecting the activity and persistence of CAR-T cells.

### 7.6 Autologous Vs. Allogeneic

Although most of the clinical studies testing CAR T-cells depended on autologous T-cells, these therapies presented several limitations. The patient’s cell generation is a cost-time-consuming process that holds a risk of manufacturing failure (Zhao J. et al., 2018). Additionally, it might result in a delayed availability of treatment, which could be problematic for patients with aggressive and highly proliferative diseases (Depil et al., 2020). The patients usually receive lymphodepleting chemotherapy, which might affect the quality and quantity of the starting autologous T cells (Ceppi et al., 2018); in contrast, allogeneic CAR-cells (derived from healthy donors) offer fully functional cells in high amounts allowing multiple generations of “off-the-shelf” CAR T cells products (Zhao J. et al., 2018; Depil et al., 2020). The heterogenic nature of tumor cell antigen expression and the immune evasion mechanisms developed by tumor cells require CAR T-cells with multiple antigen specificities (Walsh et al., 2019). This issue could be overcome by allogeneic T-cells capable of generating several CAR T-cells products with various antigen specifiers (multivalent), unlike autologous T-cells that are known to be capable of generating (monovalent) CAR T-cells (Martínez Bedoya et al., 2021). Allogeneic CAR T-cells can be obtained from several sources such as mononuclear cells from the peripheral blood of healthy donors that are capable of providing high numbers of fitter cells than the ones derived from the patients’ blood as they have been subjected to radio- or chemotherapy (Depil et al., 2020). Umbilical cord blood is another source. Furthermore, adult somatic induced pluripotent stem cells (iPSC) can be produced by introducing specific transcription factors (Papapetrou, 2016) (Figure 2). Despite the advantages of allogeneic CAR T-cells, some limitations prevent their use in the CAR T-cells field. The first limitation is the graft-versus-host disease (GVHD) and the allo-rejection produced by the host immune cells, which would hinder the cells’ anti-tumor activity (Martínez Bedoya et al., 2021). Changes within the design of the allogeneic CAR T-cells could overcome the GVHD; these changes include the employment of genetic engineering tools such as Zinc finger nucleases (ZFN), transcription activator-like effector nucleases (TALEN), and CRISPR/Cas9, which can be utilized in knocking-out T-cell receptor (TCR) and in attenuating the GVHD. Strategies to mitigate allorejection are being evaluated; chemo-resistant CAR T-cells are also being repeatedly tested through several rounds of administration to allow more profound or prolonged lymphopenia (Poirot et al., 2015; Valton et al., 2015). Overcoming the limitations of both autologous and allogeneic CAR T-cells is a great challenge but not impossible in such a fast-growing field.

## 8 Conclusion

The employment of adaptive immunity in treating chronic and malignant diseases has been the focus of many studies over the past few decades. The CAR T-cell revolution has changed the landscape of conventional therapies used in cancer and has provided new opportunities to test these technologies against other diseases. However, CAR T-cell therapy has few limitations, slowing its widespread clinical application as a routine treatment. To overcome these limitations, various *in vivo* and *in vitro* studies have suggested innovative strategies to enhance the efficacy of CARs against blood cancers and solid tumors. Several factors have been designated as necessary in CAR T-cell design, including tumor antigen expression levels on malignant cells, the affinity of the antigen-binding domain to the target epitope, tumor burden, and costimulatory elements of CARs. However, there is still a need to elucidate and resolve the issues associated with this intriguing technology. Therefore, further development of eccentric strategies to reduce CAR T-cell therapy limitations while maintaining antitumor efficacy, cellular persistence, and expansion will be necessary to magnify the clinical applications of this therapy. Notably, “off-the-shelf” CAR-T cell products with CRISPR-Cas9 genome-edited changes to manage toxicities and persistence will hold much promise. Additionally, the utilization of synthetic biology and cell engineering technologies might break the barriers impeding allogeneic CAR T-cells from being used as universal CAR T-cells, which could be pivotal in enhancing therapeutic outcomes and overall patient survival.
